# Relationships between internalized stigma and depression and suicide risk among queer youth in the United States: a systematic review and meta-analysis

**DOI:** 10.3389/fpsyt.2023.1205581

**Published:** 2023-07-20

**Authors:** Denise Yookong Williams, William J. Hall, Hayden C. Dawes, Ankur Srivastava, Spenser R. Radtke, Magdelene Ramon, D. Bouchard, Wan-Ting Chen, Jeremy T. Goldbach

**Affiliations:** ^1^School of Social Work, University of North Carolina, Chapel Hill, NC, United States; ^2^Brown School of Social Work at Washington University, St. Louis, MO, United States

**Keywords:** LGB, queer, transgender, sexual minority, internalized stigma, internalized homophobia, youth, adolescents

## Abstract

**Background:**

Queer youth experience high rates of depression and suicidality. These disparities stem from stigma-based stressors, including *internalized stigma* (i.e., negative social views that minoritized individuals internalize about their own identity). Given the importance of this factor in understanding mental health disparities among queer youth, we completed a systematic review and meta-analysis examining the relationships between internalized stigma and outcomes of depression and suicide risk (i.e., suicidal ideation, non-suicidal self-injury, and suicidal behavior).

**Methods:**

We followed the PRISMA standards. Six bibliographic databases were searched for studies in the United States from September 2008 to March 2022. Dual independent screening of search results was performed based on a priori inclusion criteria.

**Results:**

A total of 22 studies were included for data extraction and review. Most studies examined general internalized homophobia, with few examining internalized biphobia or transphobia. Many studies examined depression as an outcome, few studies examined suicidal ideation or behavior, and no studies examined non-suicidal self-injury. Meta-analyses model results show the association between general internalized queer stigma and depressive symptoms ranged *r* = 0.19, 95% CI [0.14, 0.25] to *r* = 0.24, 95% CI [0.19, 0.29], the latter reflecting more uniform measures of depression. The association between internalized transphobia and depressive outcomes was small and positive (*r* = 0.21, 95% CI [−0.24, 0.67]). General internalized queer stigma and suicidal ideation had a very weak positive association (*r* = 0.07, 95% CI [−0.27, 0.41]) and an even smaller, weaker positive association with suicide attempt (*r* = 0.02, 95% CI [0.01, 0.03]).

**Conclusion:**

Implications for clinical practice, policy, and future research are discussed.

## Introduction

In the United States, approximately 10% of the adolescent population (ages 13–17 years) self-identify as queer (e.g., lesbian, gay, bisexual, transgender, non-binary, queer+) (1). Queer youth experience higher rates of adverse mental health outcomes [e.g., depression and suicidality; (2–4)] than their heterosexual and cisgender counterparts. Minority stress theory posits that these mental health disparities are primarily due to stigma-based stressors (5, 6), such as *internalized stigma*, or negative social views about a socially marginalized minority group which individuals internalize about their own identity (7, 8). This systematic review and meta-analyses updates and expands upon a systematic review and meta-analyses from Newcomb and Mustanski (8), who reviewed research up to August 2008 between internalized homophobia and its links to mental health outcomes in sexual minorities. In our review, we synthesize evidence from September 1, 2008, to March 1, 2022, on the relationships between internalized stigma and depression and suicide risk among queer youth (including gender diverse youth) to update what is currently known to better inform research and interventions for these populations. Though we categorize queer youth as an umbrella term to embody both sexual and gender minorities in this manuscript unless specifically noted, it is important to acknowledge that sexual orientation and gender identity are two disparate identities.

### Depression, non-suicidal self-injury, and suicide risk among queer youth

Results from the Youth Risk Behavior Survey (YRBS), a nationally representative school-based survey of U.S. high school students, show disproportionately high rates of mental health problems among queer youth, including depression, suicidal ideation, and suicide attempt (9). These mental health disparities are also heightened among subgroups of queer youth, in particular transgender, non-binary, bisexual, pansexual, and queer-identifying youth. YRBS data showed that 32% of cisgender, heterosexual youth felt sad or hopeless almost every day for at least 2 weeks vs. 53–66% of queer youth (9). Suicidal ideation and suicide attempt statistics are also heightened among queer youth. In the last year, 15% of heterosexual cisgender youth seriously considered suicide, compared to 44–47% of queer youth. Six percent of heterosexual, cisgender youth attempted suicide in the past year, compared to 23–29% of queer youth (9).

Compared to other age groups, adolescents engage in higher rates of non-suicidal self-injurious behaviors (NSSI; e.g., cutting, bruising, and burning) to cope with psychological distress rather than with an intent to end one's life; the associations between NSSI and depression, suicidal ideation, and suicidal behaviors cannot be ignored and are included in our systematic review (10–13). Though limited, emerging research suggests that queer youth have a higher rate of NSSI behaviors than cisgender, heterosexual youth. In a systematic review and meta-analysis on prevalence of NSSI among queer youth, lifetime rates were elevated among sexual (30%), and gender (47%) minority individuals compared to heterosexual and/or cisgender peers (15%). Notably, among queer youth subgroups, lifetime rates of NSSI were higher among bisexual (42%) and transgender youth [47%; (14)]. It is likely these mental health disparities are higher to-date. In a systematic review investigating mental health among queer populations in the context of the global coronavirus disease-19 (COVID-19) pandemic, findings suggest heightened rates of depression, and psychological distress among queer people, largely due to the impact of systemic oppression and minority stress on mental health (15).

### Minority stress theory

Minority stress theory (5, 16) is one of the most predominant theoretical models used to examine and explain mental health disparities among queer people. A key tenet of the theory is that queer people face socially based stressors because of their stigmatized minority identity, in addition to typical life stressors (e.g., injury, financial difficulties, and relationship challenges). Consequently, the burden of stigma-based, minority-specific stressors, and societal oppression leads to increased rates of mental health problems among queer people. Specific stressors outlined in minority stress theory include violence, victimization, social rejection, and discrimination. Other stressors are more proximal in nature and include expectations of negative, prejudice-based experiences, decisions and struggles around concealing one's queer identity amid social hostility, and internalized stigma.

### Internalized stigma among queer people

*Internalized stigma* occurs when members of a minoritized group, such as queer people, cognitively encode, and maintain negative information about their identity from social environments (17, 18). Given the pervasiveness of heterosexism and cisgenderism, queer youth can perceive negative socio-cultural messages and information about queer people from an array of contexts, including their families, schools, peer groups, religious groups, communities, and exposure to mass media and social media (19). The content of such messages and information can include themes of queerness as abnormal, unhealthy, unnatural, psychopathological, and harmful (19). Internalized stigma can manifest as negative attitudes, beliefs, and affect about queer identities, relationships, and communities. Internalized stigma in queer people has been a focus of scholarship over the last 4 decades. The frequency of the term “internalized homophobia” in published literature by year escalated during the 1980s and has remained high since the early 1990s (20). Internalized stigma among queer people has been described in the literature using various terms [e.g., internalized homophobia, internalized homonegativity, internalized heterosexism, internalized biphobia, internalized transphobia; e.g., (21–23)]. Though many studies refer to internalized stigma broadly as “internalized homophobia” in the queer community, when discussing internalized stigma among all sexual and gender identity populations, our manuscript henceforth will refer to *general internalized queer stigma* for inclusivity and uniformity. *Internalized stigma* and other types of specific stigma (e.g., *internalized transphobia* or *internalized biphobia*) will refer to study outcomes that delineate between the specific types of stigmas affecting sexual and gender minority youth.

### Internalized stigma and mental health outcomes

Three evidence syntheses have been published that examined relationships between internalized stigma and mental health outcomes among queer people (8, 24, 25). In Williamson (25) critical review of the empirical literature, three studies were noted that found significant positive correlations between internalized stigma and depression. Only two studies were reviewed regarding internalized stigma and suicidality, yet both found an important connection. Szymanski et al. (24) also conducted a critical literature review of studies examining internalized stigma and depression and psychological distress, which included 15 studies of sexual minorities published between 1986–2006. They found significant associations between internalized stigma and the following outcomes: depression among sexual minority women (*r* = 0.28), depression among sexual minority men (*r* = 0.35), depression among sexual minority men and women (*r* = 0.14), psychological distress among sexual minority women (*r* = 0.29), and psychological distress among sexual minority men (*r* = 0.41). Only one study in this review examined the relationships between internalized stigma and suicidal ideation and found an overall moderate correlation (*r* = 0.29). Newcomb and Mustanski (8) completed a meta-analytic review of 31 studies using data collected during 1986–2008 with lesbian, gay or bisexual (LGB) participants and mean sample ages ranging from 17–68 years; 8 of the 31 studies were youth samples. This review found a moderate correlation between internalized homophobia and internalizing mental health problems (i.e., symptoms of depression and anxiety; *r* = 0.27). They also found that age moderated the relationship between internalized stigma and internalizing problems with stronger associations between the variables as sample ages increased. Further, the associations between internalized stigma and depression were stronger than the associations between internalized stigma and anxiety. Participant sex/gender was not a significant moderator.

A systematic review of studies examining internalized stigma and mental health outcomes has not been published since Newcomb and Mustanski (8), which included data collected up to 2008. Since 2008, research on this topic has continued to expand in terms of number of studies and researchers examining various forms of internalized stigma among queer people (e.g., internalized homophobia, biphobia, and transphobia), however, this research has not been evaluated or synthesized. There have been considerable social changes in the United States over the past 10–15 years in terms of social threats, discrimination, advancements in health and mental health care, and some legal protections regarding queer people (26–29); therefore, it is important to consider the role of internalized stigma in the lives of queer people in recent years. In addition, the prior reviews primarily focused on adults rather than youth. Given developmental issues specific to youth [e.g., identity development; (30, 31)], age-related differences in the association between internalized stigma and mental health (8), and the disproportionately high rates of depressive symptoms and suicidality facing queer youth (9), there is a need for a focused review on this particular age group. Prior reviews also primarily focused on gay and lesbian people, with limited attention to other identities within queer communities that have increased in visibility in recent years, such as bisexual, pansexual, transgender, and gender non-conforming people (32, 33). Another gap in the literature is the limited focus on the relation between internalized stigma and suicidality. Given that suicidality is a pressing public health issue in queer communities and frequently connected with depression in youth and young adults (34–36), the need to examine suicidality outcomes is clear.

### Purpose of the review

This systematic review seeks to build upon the findings from prior evidence syntheses (8, 24, 25) by focusing on queer youth in the United States ages 10–21 years and examining the relationships between internalized stigma and depression and/or suicide risk in this particular population. In the context of the Population, Exposure, Comparator, Outcome (PECO) framework (37), our review seeks to answer a multifaceted research question: How is internalized stigma (E) among queer youth (P) in the United States related to the mental health outcomes (O) of depression and suicide risk (i.e., NSSI, suicidal ideation, suicidal planning, and suicide attempt)? Additional information related to the PECO question and operationalization of these concepts are outlined in the research protocol (Appendix A).

## Methods

The preparation of this systematic review followed guidelines outlined in the Preferred Reporting Items for Systematic Reviews and Meta-Analyses (PRISMA) 2020 criteria (38), with equity-focused considerations as outlined by Welch et al. (39). A detailed research protocol was created a priori; though the protocol was not registered on PROSPERO, it is available in Appendix A. As this study incorporated findings from published literature, no ethical review process was required.

### Inclusion criteria

Studies were included in the review if they met the following criteria: (a) written in English; (b) occurred in the United States; (c) published or written since September 2008; (d) had a mean sample age between 10 and 21 years; (e) measured participants' queer identity (e.g., gay, lesbian, bisexual, transgender, queer+); (f) measured internalized stigma as an independent variable using validated self-report measures; (g) measured internalizing depressive symptoms, a depressive disorder diagnosis, and/or suicide risk (i.e., NSSI, suicidal ideation, suicidal planning, suicide attempt) as a dependent variable using validated self-report measures or clinical diagnostic interview questions; and (h) reported quantitative results on the associations between internalized stigma and depression and/or suicide risk among queer youth. A measure was considered validated if it had evidence of content validity and an additional form of measurement validity (e.g., criterion validity and predictive validity).

Studies conducted outside of the United States were excluded to ensure that socio-historical-political contexts were similar across youth populations. As various countries and nations have differing cultural values, norms, and social constructions related to sexual orientation and gender identity, we wanted to keep the contexts as uniform as possible across studies in the review. As this review is an update and expansion from prior reviews, which included literature up to August 2008 (8), we included studies since September 2008. Though some evidence indicates the prevalence of suicidal ideation may be rising and occurring in elementary-aged youth (40), depression and suicidality among pre-pubescent youth below the age of 10 is still rare (41, 42). The sample age requirement of 10–21 years was intended to prioritize youth populations (including adolescents and emerging adults) that experience higher rates of depression and suicidal risk, while considering the transitional age of 21 for some state foster care/child welfare systems, in which queer youth are heavily represented (43, 44). The World Health Organization categorizes “young people” starting at age 10 years (45), and suicide is a leading cause of death in the United States among youth in this age range (46). Additionally, the 10–21-year age range is a different developmental period; the majority of youth have reached the major queer identity milestones by age 21 years (30).

### Search procedure

A behavioral and social sciences librarian was consulted during the search process to assist with development of search strings and identifying relevant computerized bibliographic databases. For studies published in English since September 1, 2008, uniform search strings were utilized in all six databases as outlined in the search strategy (see Appendix A). Databases included APA PsycInfo, PubMed, ProQuest Dissertations and Theses Global, Sociological Abstracts, Social Services Abstracts, and Cumulative Index to Nursing and Allied Health Literature (CINAHL), as well as a review of the first 50 search results of Google Scholar sorted by relevance. Multiple databases were searched to increase the likelihood that researchers would identify all possible studies within the scope of this review, including gray literature (e.g., dissertations) to reduce the threat of publication bias.

On March 1, 2022, all searches were performed in each database and the academic search engine Google Scholar. Key search terms were entered into the PsycInfo and CINAHL databases via the EBSCO platform within multiple fields: titles, abstracts, keywords, and subject headings. Databases explored via ProQuest (i.e., ProQuest Dissertations & Theses Global, Sociological Abstracts, Social Services Abstracts) searched for key terms in all fields except for the full text (e.g., title, abstract, author, subject). PubMed was searched with terms searched within titles, abstracts, and subject headings. The same search strings and Boolean operators were used in each search, as follows: (“internalized stigma” OR “internalized homophobia” OR “internalized homonegativity” OR “internalized biphobia” OR “internalized binegativity” OR “internalized transphobia” OR “internalized transnegativity” OR “internalized heteronormativity” OR “internalized heterosexism” OR “internalized sexuality stigma” OR “self-stigma” OR “internalized sexual stigma”) AND (“sexual minority” OR “gender minority” OR queer OR LGBTQ OR “lesbian/gay/bisexual/transgender/queer” OR homosexual OR pansexual OR LGBT OR GLBT OR “sexual minorities” OR “gender minorities” OR lesbian OR gay OR bisexual OR transgender OR queer) AND (depress^*^ OR “internalizing symptoms” OR suicid^*^ OR “self-injury” OR “self-injurious behaviors” OR “psychological distress”) AND (youth OR adolescent^*^ OR teen^*^).

### Screening methods

After performing the database searches, results were imported into Sciwheel, a citation management software program, to assist with organization and duplicate removal. For reference, Appendix B depicts the PRISMA flowchart from identification, screening, and inclusion of studies. Following Sciwheel's automated duplicate removal, remaining studies were subsequently transferred to Covidence, a web-based software platform that assists with streamlining the production of systematic reviews. Additional duplicate studies were removed after the transfer to Covidence, with the remainder to screen. A checklist of inclusion and exclusion criteria was created prior to the search and used for screening in Covidence to assess study eligibility. The first author and a trained co-author (SRR) independently screened each study to determine eligibility. During the initial screening of 219 titles and abstracts, there were only three disagreements between the screeners. To resolve conflicts, screeners met to discuss and review conflicts, reaching a consensus decision on whether to include the study-−194 studies were deemed irrelevant. During this process, a third trained screener (WC) was available for consultation to assist in resolving conflicts with a thorough discussion. To examine inter-rater agreement for screening of the titles and abstracts, the decisions of the two screeners were compared and Cohen's kappa statistics were calculated with SPSS (version 21), which showed good agreement (47): kappa = 0.87, *p* ≤ .01.

In the second stage of screening, 25 full texts were reviewed by both screeners (DYW, SRR) to determine if the studies met all the inclusion criteria. During the full-text screening, an additional three studies were excluded, two of which were duplicates. The remaining study was discussed between two trained screeners (DYW, WJH), who agreed that it did not meet criteria for measurement of suicide risk. After completing search and screening processes, 22 studies were included for extraction and review, as depicted in the PRISMA 2020 flowchart depicted in Appendix B.

### Data extraction

A data extraction spreadsheet was created in Excel to identify and collect relevant information from the 22 included studies for this review. Information extracted included the citation, sample age, study design, sampling strategy, sample size, location and year data were collected, sample characteristics (e.g., sexual orientation; ethnicity/racial identity; and gender identity), measurements of internalized stigma, measurement of depression or suicide risk, analyses performed, and results regarding the relationships between internalized stigma, depression, and/or suicide risk. The first author was the main extractor of this information, and a trained co-author reviewed (SRR) the extraction data for accuracy, with both parties agreeing on the final spreadsheet. A third trained co-author (WC) also reviewed the final spreadsheet.

### Methodological appraisal

The research team initially met and discussed possible tools to evaluate the methodological quality or risk of bias for each study. To strengthen robustness of methodological appraisal, research team members (DYW, WJH) worked with co-author (MR) to modify the Joanna Briggs Institute (JBI) Checklist for Analytical Cross-Sectional Studies, a critical appraisal tool for systematic reviews (48). The modified checklist created by a co-author (MR) with input from additional researchers (DYW, WJH) removed question four regarding standard criteria used for measurement of depression as a clinical diagnosis was not criteria for these studies. Questions five and six in the original checklist were modified as these articles are not intervention studies; rather, studies with analyses that included confounders for internalized stigma (the exposure) that could be controlled for in a multivariate analysis were considered. The research team added a question related to a study's design as longitudinal or cross-sectional. Two reviewers (MR, DYW) independently evaluated the risk of bias for each study and assigned scores based on the modified checklist (see Appendix C). Two disagreements arose during comparison of results, but consensus among reviewers was reached through discussion and reviewing the article text together. Sixteen studies were considered good quality with a low risk of bias [score ranges of 6 to 7 points; (49–64)]. Six studies were of fair quality with a moderate risk of bias [i.e., range of scores from 3 to 5.5, (65–70)].

### Data synthesis

#### Narrative synthesis methods

Initial review of the included studies revealed that a quantitative synthesis, such as a meta-analysis, was appropriate considering the methodological homogeneity of the studies in terms of variables, measures, and types of statistical associations reported in the findings. Additionally, a narrative thematic synthesis (71) was utilized, reviewing the design and methodological characteristics of the studies and substantive results or findings. Results were categorized according to the type of internalized stigma measured (i.e., general internalized queer stigma, internalized transphobia), measure used for depressive symptoms (i.e., Center for Epidemiologic Depression Scale [CES-D] or other), and conceptualization of the outcome suicide risk (i.e., suicidal ideation vs. suicide attempt).

#### Meta-analysis methods

Initial review of the included studies revealed that a quantitative synthesis of associations between internalized stigma and some of the measured mental health outcomes, in the form of a meta-analysis, could be undertaken. Multiple meta-analysis models were run: one model for each type of internalized stigma (i.e., general internalized queer stigma and internalized transphobia) corresponding to each outcome (i.e., depression, suicidal ideation, suicide attempt). We were unable to run meta-analysis models for NSSI and suicidal planning because there were insufficient effect size data or studies on these outcomes.

As meta-analysis can be performed with data from two studies (72), we ran meta-analysis models on general internalized queer stigma and internalized transphobia. The two studies measuring internalized biphobia (52, 53) could not be used in a distinct model for internalized biphobia because they drew data from the same sample. Therefore, data from one of these studies (52) was included in the models for general internalized queer stigma. Additionally, a subgroup analysis was run to explore heterogeneity by comparing the general internalized queer stigma and depression outcomes measured by either the CES-D or other depression symptomatology scale. We used standardized beta coefficients or correlation coefficients as effect size data, which is an acceptable strategy in meta-analysis (73). When effect size data needed for meta-analysis were reported in other formats (e.g., odds ratios), the data were transformed using formulas recommended by Lenhard and Lenhard (74).

The Meta-Essential program [version 1.5, (75, 76)], was used to run the meta-analysis models. Random-effect models were used as we assumed that the true mean effects could vary across samples and studies. An inverse variance weighting method with an additive between-studies variance component based on the DerSimonian-Laird estimator was used (75). Confidence intervals (CIs) for the mean effects were estimated using the weighted variance method for random-effect models (77), and individual study effect sizes and their CIs were calculated using the Student's t-distribution. No missing values were identified.

## Results

A total of 22 studies were included in this review: 17 peer-reviewed journal articles (77%), 4 doctoral dissertations (18%), and 1 master's thesis (5%). A summary of the methodological characteristics of these studies and a synthesis of the substantive findings regarding internalized stigma, depression, and suicide risk follows. Appendix D shows a summary of information extracted from studies related to internalized stigma and depression outcomes. Appendix E presents study summaries related to general internalized queer stigma and suicide risk. Out of the 22 studies, 1 examined both depression and suicide risk as outcomes (69); this study's findings were separated and recorded in the appropriate corresponding Appendix D (depression-related outcomes) or E (suicide risk-related outcomes), depending on the outcome variable.

### Methodological quality of the studies

#### Designs

Of the 22 studies, 14 (64%) were cross-sectional and 8 (36%) were longitudinal designs. All studies relied solely on quantitative methods and non-probability sampling (e.g., convenience, purposive, and/or snowball sampling techniques). Twelve studies (55%) sampled participants from a single city or locale in the United States (*n* = 7 for Chicago and the Mid-Western region, *n* = 3 for Boston and the Mid-Atlantic region, *n* = 1 in Memphis, *n* = 1 from Seattle). Eight studies (36%) did not specify a location, as they also included online components for out-of-state individuals for recruitment or data collection (i.e., surveys); of these, 1 (66) included some individuals from Canada, though most participants were from the United States. Only 2 studies (9%) were drawn from national samples. Recruitment sites varied across studies and included social media, school- and community-based organizations supporting queer individuals, web sites, peer referrals, gender programs within medical settings, and crisis centers supporting queer youth.

#### Samples

Sample sizes varied from 30 to 2,949 individuals, with most studies having *N* = 150–600 (50% of studies), followed by *N* < 150 (36% of studies), and 3 studies (14%) with large sample sizes of more than 600 participants. Overall, the mean of all mean sample ages is 19.06 years, and the range of the sample age means is 15.1 to 21.38 years, with the majority of studies (59%) only including individuals aged 24 years or younger. In terms of targeted sample demographics related to sexual orientation, gender identity, sex assigned at birth, and ethnic/racial identities, percentages of samples varied in range due to heterogeneity of the sample population studied. As outlined in Appendices D, and E, 4 (18%) studies focused on outcomes related to intersectional identities of both sexual and gender minority youth broadly, collecting and reporting demographic information for both identity aspects. Four (18%) studies focused only on individuals assigned male at birth (AMAB) or young men who have sex with men. Three (14%) studies focused only on LGB youth but excluded gender minority youth, and 3 studies (14%) focused only on gender minority youth. The remaining 8 studies specifically identified their sample as the following: assigned female at birth (AFAB) or young women who have sex with women (*n* = 2), only bisexual-identifying AFAB (*n* = 1), transfeminine AMAB (*n* = 1), only Black or African American sexual minority youth (*n* = 2), and only Latina/o/e-identifying sexual minority youth (*n* = 1) or Latina/o/e-identifying sexual minority and gender minority youth (*n* = 1). However, samples across the 22 studies were generally diverse in terms of sexual orientation, gender identity, and race/ethnicity. Twenty-one (96%) of the 22 studies incorporated sexual orientation demographics, 20 separated gender identity (though at times only dichotomous “male” vs. “female”), and 20 reported on racial/ethnic identity demographics. About half of the studies (45%) reported on either parent or participant education levels, and 8 studies (36%) reported sex assigned at birth. Few studies reported other participant demographics such as household income (27%), employment status (18%), religious affiliation (18%), and marital status (5%). No studies reported on ability/disability status or immigrant/citizenship status.

#### Measurement of internalized stigma

All studies used self-report scales or items to measure internalized stigma, though the type of internalized stigma measured, and scales utilized, varied between some studies. Most studies (82%, *n* = 18) measured general internalized queer stigma, some of which separated gender identity and sexual orientation whereas others did not. Additional studies specifically measured outcomes related to internalized transphobia (14%, *n* = 3), or internalized biphobia (4.5%, *n* = 1). For the studies measuring general internalized queer stigma, the measures used varied widely; 13 different scales or items were used among the 18 studies measuring general internalized stigma. The most utilized scale to measure general internalized queer stigma was the Desire to be Heterosexual Subscale (*n* = 3), followed by the Revised Homosexual Attitudes Inventory (*n* = 2). Of the remaining, several were unnamed but reported as “adapted” [i.e., the measure cited in the Bruce et al. (50, 64) studies; one unnamed subscale from Puckett et al. (69)]. Though not an exhaustive list, additional scales utilized by only one of the remaining studies include the Sexual Minority Adolescent Stress Inventory, and 2 studies created a measure of internalized general queer stigma using 3 dichotomous items (54, 67). Sixteen of the 18 general internalized queer stigma studies cited internal consistency reliabilities (i.e., Cronbach's alphas) ranging from 0.72 to 0.95. For the 3 studies examining internalized transphobia, 2 (67%) measured this concept using the Gender Minority Stress and Resilience scale (α = 0.90), whereas the remaining 1 (33%) used the Transgender Identity Survey (α = 0.94). The 1 study measuring internalized biphobia utilized the Bisexual Identity Survey (α = 0.70 to 0.85). In all 22 studies, internalized stigma was measured through a series of Likert-type scale items that were summed and treated as a continuous variable, with higher mean scores indicating higher levels of internalized stigma.

#### Measurement of depression

All studies used self-report scales or items to measure depression-related outcomes. A total of 19 studies (86%) measured depression, though 1 of these studies (69) also measured suicide risk outcomes. As such, 82% (*n* = 18) of the total studies examined only depressive symptom outcomes; however, Goldbach et al. (67) observed the variable “*psychological distress”* and operationalized this outcome by incorporating dichotomous items related to both hopelessness and suicidality. Seven of the 19 studies (37%) used the 20-item version of the CES-D and 3 studies (16%) used the 10-item version of the CES-D. Three studies measured depression using the Patient-Reported Outcomes Measurement Information System (PROMIS) Depression Short Form 8a, and 2 studies used the Brief Symptom Inventory-18. One study per scale used the Patient Health Questionnaire-9, the Adult Self-Report developed by the Achenbach System of Empirically Based Assessment, and the Youth Inventory-4. In 13 studies, depressive symptoms were measured through a Likert-type scale measurement and subsequently treated as a continuous variable in analyses. Four studies applied cutoff scores per measurement scoring recommendations, to distinguish clinically significant levels of depressive symptoms, with reported internal consistency reliability values ranging from 0.73 to 0.95.

#### Measurement of suicide risk

All studies used self-report items to assess suicide risk, though this outcome was operationalized in various ways, making comparisons between the studies difficult. Of all the studies included in our systematic review, only 4 studies (18%) measured suicide risk as an outcome, though as mentioned prior, 1 study (69) also measured depression outcomes and was included in both Appendices D and E for study summaries. Three of the 4 suicide-risk related studies (75%) asked dichotomized questions related to suicidal thoughts over time (e.g., the last month or previous 6 months), and all 4 studies asked about suicide attempts (e.g., within the last 6 months, previous year, or over their lifetime). Two studies asked explicitly if suicide attempts were related to one's queer identity, and only 1 study asked about suicide plans (58).

### Relationships between internalized stigma and depression

Fifteen studies examined the relationships between general internalized queer stigma and depression, 3 studies explored internalized transphobia, and 1 study looked at internalized biphobia. Of the 15 studies focusing on general internalized queer stigma, 73% (*n* = 11) of them found statistically significant, positive associations between internalized stigma and depression. Two of the 15 studies with longitudinal designs indicated some mixed findings; the relationship between general internalized queer stigma and depression was significantly positively correlated in some models but showed no significant association in others (60, 61). Two studies (57, 68), which were dissertations, did not find a statistically significant relationship between general internalized queer stigma and depression. However, it is important to note that Gutiérrez (68) cited conflicting findings between their study and previous research, as well as issues related to their internalized queer stigma outcome including violations of normality assumptions, positive skewness, and low levels of internalized homonegativity in the sample. Researchers indicate caution for interpretation of their results (68). All studies measuring internalized transphobia (*n* = 3); (51, 55, 56) found that this type of internalized stigma was significantly and positively associated with depression/depressive symptoms. The 1 study (53) examining stigma in only bisexual individuals determined that internalized biphobia was positively associated with depression. Among all studies examining the relationships between internalized stigma and depression, 15 out of the 19 studies (79%) found statistically significant, positive associations, 2 (11%) found both significant and insignificant associations depending on the model, and 2 found no significant relationships (11%).

### Relationships between internalized stigma and suicide risk

A total of 4 studies examined internalized stigma and suicide risk in queer youth. Only 3 studies (54, 58, 69) examined the relationship between general internalized queer stigma and suicide risk, with varying outcomes related to suicide risk (e.g., suicidal ideation, suicide planning, and suicide attempt). One additional study (66) examined *internalized self-stigma* and its connection to suicide risk; however, their study purpose explicitly focused on understanding suicide risk among transgender youth. Two studies (54, 58) observed suicidal ideation, both finding significant, positive correlations between general internalized queer stigma and suicidal thoughts over time. However, in one model, this relationship became non-significant when controlling for baseline suicidality and demographics in a gray literature publication (58). This same study looked at the relationship between general internalized queer stigma and suicide plans over a year and did not find a statistically significant relationship between the two variables (58). The 2 studies examining general internalized queer stigma and suicide attempt found that there was not a statistically significant association between general internalized queer stigma and suicide attempt; a similar relationship was found in the study only examining suicidality in transgender youth. However, the strongest predictor of reporting suicide attempt was whether respondents had lost friends after coming out as a queer individual (69). Notably, there were no articles found in this systemic review process that attempted to explore the relationships between internalized queer stigma and NSSI behaviors.

### Meta-analysis results

#### Relationships between internalized stigma and depression

Table 1 shows the summary of studies included in our meta-analyses, and Table 2 shows the results of the meta-analyses for all models run, including the following four related to depression outcomes: general internalized queer stigma and depression (*n* = 12); general internalized queer stigma and depression measured by the CES-D (*n* = 7); general internalized queer stigma and depressive outcomes measured by other scales (*n* = 5); and internalized transphobia and depression (*n* = 3). Bruce et al. (50) was excluded from the general internalized queer stigma and depression models as they drew from the same sample of Bruce et al. (78). Zhao et al. (63) was excluded from our meta-analysis models as they analyzed the same dataset as Anhalt et al. (49); authors chose to utilize the effect size from the larger analytic sample. Additionally, the first author reached out to the author to get effect size information that could be used in the meta-analysis but did not receive the necessary information; therefore, one additional study was excluded from the general internalized queer stigma and depression meta-analysis (62).

**Table 1:** Summary of studies included in the meta-analysis.

**Model category and study citation**	***n***	***r***	**[95% CI]**	**Weight (%)**
***General internalized queer stigma and depression***				
Anhalt et al. (49)^*^	377	0.27	[0.18, 0.36]	11.14
Armelie (65)^*^	65	0.27	[0.04, 0.51]	3.07
Bruce et al. (78)^*^	200	0.25	[0.11, 0.38]	7.33
Dyar et al. (52)	488	0.13	[0.04, 0.22]	11.99
Goldbach et al. (67)	1,911	0.15	[0.10, 0.19]	19.11
Gutiérrez (68)	235	0.07	[−0.06, 0.20]	7.65
Langdon (57)	86	0.21	[0.00, 0.42]	3.71
Mereish et al. (59)^*^	94	0.21	[0.01, 0.41]	4.01
Puckett et al. (69)^*^	61	0.32	[0.09, 0.55]	3.06
Sarno et al. (60)	1,130	0.25	[0.20, 0.30]	17.21
Simonson (61)^*^	135	0.19	[0.02, 0.36]	5.32
Walker and Longmire-Avital (70)^*^	175	0.16	[0.01, 0.31]	6.39
***Internalized transphobia and depression***				
Chodzen et al. (51)	109	0.02	[−0.18, 0.21]	35.83
Jackman et al. (55)	133	0.30	[0.14, 0.46]	40.29
Katz-Wise et al. (56)	30	0.37	[0.04, 0.70]	23.88
***General internalized queer stigma and suicidal ideation***				
Gibbs and Goldbach (54)	2,949	0.06	[0.02, 0.09]	77.91
Lawlace (58)	369	0.12	[0.02, 0.22]	22.09
***General internalized queer stigma and suicide attempt***				
Gibbs and Goldbach (54)	2,949	0.02	[−0.02, 0.05]	87.47
Lawlace (58)	369	0.02	[−0.08, 0.13]	10.90
Puckett et al. (69)	61	0.04	[−0.23, 0.31]	1.64

^*^Denotes the study utilized The Center for Epidemiological Studies-Depression (CES-D) scale to measure depression.

**Table 2:** Meta-analysis modeling results.

**Model**	***N***	***k***	***r***	**[95% CI]**	***T***	***I^2^ (%)***	***Q***	***p_*Q*_***
General internalized queer stigma and depression	4,957	12	0.19	[0.14, 0.25]	0.04	43.09	19.33	0.06
*Measure of depression: CES-D*	1,107	7	0.24	[0.19, 0.29]	0.00	0.00	2.57	0.86
*Other measures of depression*	3,850	5	0.16	[0.08, 0.26]	0.06	68.54	12.71	0.01
Internalized transphobia and depression	272	3	0.21	[-0.24, 0.67]	0.15	66.68	6.00	0.05
General internalized queer stigma and suicidal ideation	3,318	2	0.07	[-0.27, 0.41]	0.00	27.88	1.39	0.24
General internalized queer stigma and suicide attempt	3,379	3	0.02	[0.01, 0.03]	0.00	0.00	0.03	0.99

After running the initial meta-analysis model for general internalized queer stigma and depression, substantial heterogeneity was noted. One heterogeneity issue noticed was the different measures used for outcomes; for example, though numerous studies used the CES-D to measure depression, other studies used lesser-known measures. We investigated heterogeneity by performing a subgroup analysis with one group of studies that used the same measure (CES-D) and the other group of studies using other measures of depression (see Table 2). First, during an initial assessment, one model for general internalized queer stigma included all 12 studies on general internalized queer stigma and depressive symptoms and produced a positive association between general internalized queer stigma and depressive symptoms (*r* = 0.19, 95% CI [0.14, 0.25]). Additional analyses were run to partition the studies utilizing the CES-D measures against the others, and results were compared. For those 7 studies with the uniform measures of depressive symptoms, our subgroup analysis revealed a slightly stronger positive correlation between internalized queer stigma and depression (*r* = 0.24, 95% CI [0.19, 0.29]). Subgroup analyses were also analyzed by combining the studies measuring internalized transphobia specifically. The association between internalized transphobia and depressive outcomes was small and positive (*r* = 0.21, 95% CI [−0.24, 0.67]).

#### Relationships between internalized stigma and suicide risk

Two models were run to explore the relationships between general internalized stigma and suicide risk, specifically with the outcomes of suicidal ideation (*n* = 2) and suicide attempt (*n* = 3). The fourth study (66) was omitted as this was the only suicide risk-related outcome study that pulled from a sample of strictly transgender youth. Meta-analyses model results in Table 2 showed that general internalized queer stigma and suicidal ideation had a very weak positive association (*r* = 0.07, 95% CI [−0.27, 0.41]). General internalized queer stigma and suicide attempt had a smaller, weaker positive association (*r* = 0.02, 95% CI [0.01, 0.03]).

Following the meta-analyses on the relationships between internalized stigma and depressive symptoms, researchers utilized Egger's regression test to assess for publication bias (79). Egger's test for a regression intercept resulted in *t*_(12)_ = 0.68, *p* = .51, indicating no evidence or low risk of publication bias. For suicide risk-related outcomes, there were an insufficient number of studies (<10) to assess for publication bias; as such, the publication bias test results are not reliable.

## Discussion

Findings from this review suggest that there are differences in terminology and use of measures to assess internalized stigma among queer samples. Most of the studies focused on general internalized homophobia or queer stigma, with a few assessing internalized transphobia and biphobia, implying a lack of research focus on diverse forms of internalized stigma among queer populations (80, 81). Only focusing on general internalized queer stigma may fail to capture specific experiences of all queer people, such as bisexual, pansexual, transgender, and non-binary people (26, 27, 82). In terms of measurement, 13 measures of general internalized queer stigma were used in 16 studies, two measures of internalized transphobia were used in three studies, and only a single study measured internalized biphobia. Prior research has acknowledged that comparison of internalized stigma measures is complicated because of the lack of studies using different measures with same population, differences in the outcomes measured, and insufficient understanding of which subscales or dimensions of the measure corresponds with the studied outcome (24, 83, 84). Further work is needed to evaluate the conceptual issues around definition, operationalization, and measurement across multiple measures of internalized stigma and responsiveness toward inclusion regarding sexual and gender identity diversity and intersectional experiences among those with multiple minoritized identities (85–88).

Findings on the association between internalized stigma and depression and suicide were mixed. Over 70% of the studies reported a positive association between general internalized queer stigma and depression, while there was a positive association among all studies examining internalized transphobia and biphobia. However, we found more variation in associations between internalized stigma and suicide risk by outcome (i.e., ideation, attempts, and plans). Meta-analysis results revealed a small association between general internalized queer stigma and depression (*r* = 0.19 to 0.24 with mixed or similar depressive symptom measures), but low/weak associations between internalized stigma and suicide risk (i.e., suicidal ideation [*r* = 0.07]; suicide attempt [*r* = 0.02]). Our finding on the association between internalized stigma and depression is similar to findings from past reviews (8, 24), although our mean effect size is slightly smaller. It may be that as U.S. society has generally become more accepting of queer people in recent time (89) and more queer-specific psychosocial supports and community resources have become available to queer youth, the link between internalized stigma and depression has weakened. Another noteworthy finding was the associations between internalized stigma and depression were stronger than the associations between internalized stigma and suicide risk. We believe the low/weak positive relation between internalized stigma and suicide risk could be because of a mediating effect of depression on the association between internalized stigma and suicidality (90, 91). For example, a review found that as many as 60% of suicide deaths and 40–80% of suicide attempts occur among young people with depression (92). Our analysis found a similar pattern; however, studies are needed with standardized measures on outcome variables for comparison and uniformity.

Findings from our review are in line with the minority stress theory, a leading theoretical model for understanding mental health disparities among queer people (5, 16). Internalized stigma (proximal stressor) among queer people reflects internal or subjective response to both direct and indirect experiences within a heterosexist and prejudicial society, that can negatively impact their mental health (5, 25). Our analysis furthers support for minority stress theory; where we found a positive association between internalized stigma and depression, as indicated in prior reviews (8, 24, 25). However, across reviews, there remains limited understanding on the impact of internalized stigma on suicide risk. More research is needed to expand the understanding of the role of internalized stigma on health risks (beyond internalizing mental health disorders) among queer people. The minority stress theory model may need to be adapted for the outcome of suicidality among queer youth to illustrate factors, mechanisms, processes, and pathways for this outcome with this population group [e.g., the Queer Prevention of Youth Suicidality Model; Queer-PRYSM; (93)].

### Strengths and limitations of the review

This review followed the rigorous PRISMA standards for systematic reviews and meta-analysis, which included use of an expert-informed search string, searches of six databases, and dual independent screening. Thematic findings were complemented with meta-analysis results. The focus on a specific age group (i.e., youth) was advantageous because prior research shows that internalized stigma varies by age (8) and youth is a distinct and formative developmental period. This review was also inclusive of various identities within the umbrella of queer youth by including studies of cisgender LGB youth; transgender youth; and mixed groups of cisgender, transgender, and non-binary LGBTQ+ youth. The mental health outcomes in the review were selected because they are disparities for queer youth; therefore, this review has public health significance.

We were surprised to have only found 22 studies on this topic area given the lengthy time window (2008–2022) and pressing significance of mental health disparities facing queer youth. Unfortunately, there were insufficient numbers of studies to perform certain meta-analyses for certain subgroups of queer youth (e.g., internalized biphobia and depression) and with certain outcomes (e.g., internalized stigma and suicidal planning). Indeed, there were few studies focused on suicidality outcomes and no studies on NSSI. Another issue among the studies was that methodological heterogeneity was moderately high in certain ways, including various measures used for internalized stigma and mental health outcomes.

### Methodological considerations of the studies reviewed

Systematic reviews summarize what is substantively known about a topic and provide for critical appraisal of the state of the research on a topic. Based on our appraisal of the methodological characteristics of the studies, we identified several strengths. At least 36% of the studies were of longitudinal design, offering unique advantages in detecting changes or developments over time in regard to the outcomes of interest (e.g., internalized stigma, depressive symptoms, and suicidality). The studies were generally more balanced in terms of sex assigned at birth and gender identity (e.g., males vs. females) and incorporating individuals of different racial and/or ethnic backgrounds. Historically, LGB+ identity research has tended to focus on gay men, especially White gay men, more than other subgroups of the queer community (94, 95). Additionally, the majority of studies either ran and reported both bivariate and multivariate analyses in their research or focused specifically on complex multivariate analyses to strengthen their findings.

Conversely, several prominent methodological limitations were identified among the studies. The majority of studies utilized non-probability sampling approaches; only two studies included national samples. Additionally, some studies reflected issues related to sampling and demographics reporting related to intersectional identities (e.g., not reporting on disability status; combining or conflating sexual minority and gender minorities; treating gender identity as a binary variable). As such, we are unable to consider nuances of subgroups within larger groups despite knowing that disparities exist. Measure quality was also an area of weakness among the studies included in this review, especially as related to suicide outcomes, where heterogeneity and a dearth of research in this area made it difficult to compare studies for powerful analyses.

### Implications for practice, policy, and systems

Due to the importance of the relationships between internalized stigma and mental health, there are critical implications for practice, policy, and social systems, such as schools and mental health service environments. Affirming mental health services that could target internalized stigma can be challenging to access for queer populations due to cost prohibition, lower rates of health insurance, anti-queer service provider bias, and youth wishing to access services without their parents' knowledge. Internalized queer stigma as a concept has only existed for just over 40 years and may only recently have penetrated the knowledge base of mental health practitioners as a primary driver of queer mental health problems and important psychosocial concern among queer clients/patients (96). Consequently, internalized stigma-related sequelae, such as negative cognitions (e.g., negative self-concept) and affect (e.g., shame), are often under-examined and under-addressed within treatment settings by providers and evidence-based practices, thus, neglecting a crucial component in targeting queer youth mental health inequities.

School settings are also vital to cultivating positive queer youth development. Research shows that gay-student alliances, and queer student unions, provide positive narratives that mitigate the harms caused by internalized stigma and damaging societal messaging (97, 98). Both milieus—school and mental health settings—must go further to address internalized stigma related to queer youth having multiple minoritized identities. Educational and mental health practices often fail to consider the unique *intersectional stigmas* (i.e., stigma experienced by people with various minority statuses) faced by queer youth who may also be disabled or a racial and gender minority. Thus, not considering clinically essential treatment components. Educational and mental health treatment policy on multiple societal levels (e.g., agency/school, state, and federal) must target internalized stigma within queer-specific programming as part of comprehensive queer-inclusive plans and strategies. Policies should highlight the need for affirming school counselors, and queer-inclusive curricular resources (e.g., textbooks, classroom resources), in addition to supporting gay-student alliances and queer-student unions (99). Mental health systems and similar public systems, such as libraries, should have policies that affirm the queer youth. All these efforts should be allocated and leveraged to promote positive queer youth identity development to prevent and curb queer youth depression and suicide.

### Future research

Future research efforts should focus on measurement of these constructs (e.g., suicidality, internalized stigma) and their derivatives (e.g., suicidal behavior, suicidal ideation, internalized biphobia, and internalized transphobia), and the identification of critical confounding variables that may impact the outcomes of depression and suicidal behavior, and suicidal ideation. Further examination of internalized biphobia, and internalized transphobia as correlates to these outcomes is needed to prioritize the bisexual and transgender individuals experience as research shows have elevated risk among queer suicidal behavior (66, 100, 101). All studies observed internalized stigma as a continuous variable, however, dichotomizing between low vs. high levels of internalized stigma may assist in further analysis and understanding of these phenomena. As the studies within this review showed a relatively low or weak association between these key relationships, it is important that future research identifies possible confounding variables with the aim of creating a more comprehensive depiction as to what constructs are driving negative outcomes. Mental health disparities related to victimization, depression, and suicide also exist at the intersections of sexual orientation and racial/ethnic identity in queer youth. Current trends indicate suicidal behaviors such as suicidal ideation and suicide attempts are rising in youth of color (102, 103). While this review did not assess differences by other indicators (e.g., sex assigned at birth, racial/ethnic identity), these unique demographics should also be collected and considered during analyses when exploring both risk and protective factors. Moreover, as suicide risk is shaped by age and developmental histories more longitudinal methods measuring these variables over time for this population is needed. Considering the complex identities and experiences of queer youth in the United States, more attention must be given to this unique population to bolster protective factors and mitigate negative mental health outcomes such as internalized stigma, depression, and suicidality.

## Data availability statement

The original contributions presented in the study are included in the article/Supplementary material, further inquiries can be directed to the corresponding author.

## Author contributions

DYW was involved in all areas of the systematic review process, including meta-analysis, synthesis, and manuscript writing. WH provided substantial contributions to the analysis and interpretation of data, including meta-analysis, drafting, and revising many parts of the manuscript. HD and AS assisted in drafting a couple parts of the manuscript, along with MR and DB. MR also created the modified checklist to assess study bias, rated and scored the studies included in the review, met with the first author to discuss study bias and quality, and assisted in manuscript revisions. SR and W-TC provided assistance in the screening of articles and data extractions, as well as help in revising the manuscript. JG provided supportive assistance with the work and provided feedback on the content for publication. All authors contributed to the article and approved the submitted version.
