# Supplementary material for: Relationships between internalized stigma and depression and suicide risk among queer youth in the United States: a systematic review and meta-analysis
Source: Front Psychiatry. 2023 Jul 20;14:1205581. doi: 10.3389/fpsyt.2023.1205581 (PMC10399219; doi:10.3389/fpsyt.2023.1205581)
Supplement: Supplementary file 1 [file Data_Sheet_1.docx]

**Appendix A**

**Title:** Relationships Between Internalized Stigma and Depression and Suicide Risk among Queer Youth in the United States: A Systematic Review and Meta-Analysis

**Conceptual or Theoretical Frameworks Guiding the Review:** Minority Stress Theory; Intersectionality

**Research Questions:**

- Among queer youth ages 10-21 years in the United States, how is internalized stigma related to depressive symptoms?
- Among queer youth ages 10-21 years in the United States, how is internalized stigma related to suicide risk (i.e., suicidal ideations, self-injurious behaviors, and/or suicide attempts)?

**Participants/Population:** Sexual and gender minority (e.g., lesbian, gay, bisexual, pansexual, queer, genderqueer, transgender, nonbinary, etc.) youth populations (i.e., mean age of sample ages 10-21 years; see search terms) in the United States

**Intervention/Exposure:** Internalized stigma related to sexual orientation and/or diverse gender identity stigma (e.g., internalized homophobia, internalized transphobia; see search terms)

**Comparator:** Not applicable in our review as we are looking at the relationships between internalized stigma and depression and suicide risk rather than an intervention

**Outcome:** Depressive symptoms and/or suicide risk (e.g., self-injurious behaviors; suicidal ideation, suicide attempt; see search terms)

**Databases:**

- PsycInfo
- PubMed
- ProQuest Dissertations
- Sociological Abstracts
- Social Services Abstracts
- CINAHL
- First 5 pages (50 results) of Google Scholar

**Search Terms:**

- **For internalized stigma:** “*Internalized stigma” OR* “*internalized homophobia” OR* “*internalized homonegativity” OR* “*internalized biphobia” OR* “*internalized binegativity” OR* “*internalized transphobia” OR* “*internalized transnegativity” OR* “*internalized heteronormativity” OR* “*internalized heterosexism” OR* “*internalized sexuality stigma” OR* “*self-stigma” OR* “*internalized sexual stigma”*
- **For LGBTQ+:** “*Sexual minority” OR* “*gender minority” OR LGBTQ OR* “*lesbian/gay/bisexual/transgender/queer” OR homosexual OR pansexual OR LGBT OR GLBT OR* “*sexual minorities” OR* “*gender minorities”* *OR lesbian OR gay OR bisexual OR transgender OR queer*
- **For depression and suicide:** *Depress* OR* “*internalizing symptoms” OR suicid* OR “self-injury” OR* “*self-injurious behaviors” OR* “*psychological distress”*
- **For youth:** *Youth OR adolescen* OR teen**

**Search Plan:**

*PsycInfo via EBSCO platform:*

- Key terms entered to search within titles, abstracts, keywords, and subject headings

*PubMed:*

- Key terms entered to search within all fields (i.e., titles, abstracts, and subject headings)
- *Limiters:* Publication date on or after September 1, 2008

*ProQuest Dissertations & Theses Global via ProQuest:*

- Key terms entered to search within all fields except for the full text (e.g., title, abstract, author, subject)
- *Limiters:* Publication date on or after September 1, 2008

*Sociological Abstracts via ProQuest:*

- Key terms entered to search within all fields except for the full text (e.g., title, abstract, author, subject)
- *Limiters:* Publication date on or after September 1, 2008

*Social Services Abstracts via ProQuest:*

- Key terms entered to search within all fields except for the full text (e.g., title, abstract, author, subject)

*CINAHL via EBSCO platform:*

- Key terms entered to search within titles, abstracts, keywords, and subject headings

*Google Scholar:*

- A simplified search will be performed using some of the search terms above
- The first 5 pages (50 results) will be reviewed

**Inclusion Criteria:**

- The mean sample age was between 10 and 21 years (including months prior to age 22 years; *rationale:* Suicide is a leading cause of death in the United States amongst teenagers and young adults in this age range, sometimes ranking at 2^nd^ and sometimes at 3^rd^, 10 is when depressive disorders start coming out and externalizing at age 12—WHO also says adolescence starts at age 10 years; 21 is the time a lot of youth age out of foster care considered “transitional age youth” or “at risk youth” and is usually included in young adults/around the time youth are in college)
- Participants’ sexual orientation and/or gender identity (e.g., gay, lesbian, bisexual, queer, transgender) were measured
- Internalizing depressive symptoms or a depressive disorder diagnosis or suicide risk (i.e., suicidal ideation, non-suicidal self-injurious behaviors, or suicide attempts) were collected as a dependent variable using validated self-report measures or clinical diagnostic interview questions
- Internalized stigma was collected as an independent variable using validated self-report measures or clinical diagnostic interview questions
- Quantitative and/or qualitative results were reported on the associations between internalized stigma and depression and/or suicide risk among LGBTQ+ youth
- The study takes place in the United States
- The study was published or written from 9/2008 to present day (*rationale:* The Newcomb & Mustanski, 2010 review entitled “Internalized homophobia and internalizing mental health problems: A meta-analytic review” utilized a search up to August 2008, and there were studies with youth in there, as we operationalize youth).
- The study is written in English

**Exclusion Criteria:**

- Studies where the mean sample age is below 10 years or above 21 years and 11 months
- Participants’ sexual orientation and/or gender identity (e.g., gay, lesbian, bisexual, queer, transgender) were not measured
- Internalizing depressive symptoms or a depressive disorder diagnosis or suicide risk (i.e., suicidal ideation, non-suicidal self-injurious behaviors, or suicide attempts) were not measured
- Internalized stigma related to sexual orientation and/or gender diverse identities were not measured
- The study was conducted outside of the United States
- The study was published or written before 8/2008
- The study is written in a non-English language

**Data Extraction:** A data extraction spreadsheet will be created in Excel to identify and collect relevant information from studies for this review. Information extracted will include the article citation, sample age, study design, sampling strategy, sample size, location and year data were collected, sample characteristics (e.g., sexual orientation; ethnicity/racial identity; gender identity), measurements of internalized stigma, measurement of depression or suicide risk, analyses performed, and results regarding the relationships between internalized stigma, depression, and/or suicide risk. The first author will be the main extractor of this information, and a trained co-author will review the extraction data for accuracy, with both parties agreeing on the final spreadsheet and discussing any concerns which may arise. If consensus cannot be reached, a third trained co-author will assist in final decision-making. Additionally, the first author will e-mail primary authors of the included, relevant studies if any necessary information is missing or requires clarification.

**Appendix B**

*Flowchart Depicting the Identification, Screening, and Inclusion of Studies*

**Identification of studies via databases**

**Records removed *before screening*:**

Duplicate records removed through SciWheel and Covidence programs (*n* = 91)

**Records identified from database searching** (*N* = 310):

PsycInfo (*n* = 98)

PubMed (*n* = 93)

ProQuest Global (*n* = 17)

Sociological Abstracts (*n* = 11)

Social Services Abstracts (*n* = 5)

CINAHL(*n* = 36)

Google Scholar (*n* = 50)

**Identification**

**Records excluded** (*n* = 194)

**Records screened, title and abstract** (*n* = 219)

**Reports excluded:**

Duplicate study (*n* = 2)

Suicide risk outcome did not meet inclusion criteria (*n* = 1)

**Records screened, full text**

(*n* = 25)

**Screening**

**Reports excluded** (*n* = 0)

**Reports assessed for eligibility** (*n* = 22)

**Studies included in review**

(*n* = 22)

**Included**

*Note*. Based on the PRISMA 2020 checklist and diagram (Page et al., 2021).

**Appendix C**

*Modified Joanna Briggs Institute (JBI) Checklist for Analytical Cross-Sectional Studies*

**Reviewer Name:  ____________________**

**Date of Review:  _____________________**

**Citation: ___________________________**

|  | Yes (1) | No (0) | Unclear  (0.5) | Notes |
| --- | --- | --- | --- | --- |
| 1. Were the criteria for inclusion in the sample clearly defined? | □ | □ | □ |  |
| 1. Were the study subjects and the setting described in detail? | □ | □ | □ |  |
| 1. Was the exposure *(internalized stigma)* measured in a valid and reliable way? | □ | □ | □ |  |
| 1. Were the outcomes *(depression and/or suicide risk)* measured in a valid and reliable way OR did they use DSM criteria to measure this outcome? | □ | □ | □ |  |
| 1. Were confounding factors for the exposure (*internalized stigma)* identified that could be controlled for in a multivariate analysis? | □ | □ | □ |  |
| 1. Was appropriate statistical analysis used? | □ | □ | □ |  |
| 1. Was the study longitudinal? | □ | □ | □ |  |
|  |  |  |  | **Total Points: _____** |

**Overall appraisal:** Low Risk of Bias (High Quality, 6-7 points)

Moderate Risk of Bias (Medium Quality, 3-5.5 points)

High Risk of Bias (Low Quality, 0-2.5 points)

Include □ Exclude   □ Seek further info  □

**Comments (including reason for exclusion):**

_______________________________________________________________________________________________________________________________________________________________________________________________________________________________________________________________________________________

**Appendix D**

*Summary of Studies Included in the Systematic Review Related to Depression Outcomes (n = 19)*

| Citation | Study Purpose | Study Design, Location and Sampling Strategy | Sample Description and Response Rate | Measurement of Internalized Stigma | Measurement of Depression | Results and Findings on the Relationship(s) Between Internalized Stigma and Depression |
| --- | --- | --- | --- | --- | --- | --- |
| Anhalt et al., 2020 | To examine the impact of multiple minority stressors on adjustment outcomes of Latina/o/e sexual minority youth | Quantitative cross-sectional survey data from multiple areas across the United States using non-probability purposive sampling from social media outlets in Spanish and English | 377 Latina/o/e self-identified queer youth ages 14-24 years; *M* = 20.25, *SD* = 2.63  **Sexual orientation:** Gay or lesbian (85.1%); bisexual (7.2%); other/queer/questioning/asexual/pansexual (7.7%)  **Gender identity:** Male (74.3%); female (19.1%); transgender or nonbinary (6.6%)  **Racial or ethnic identity:** Mexican/Mexican American/Chicana/o (67.6%); Puerto Rican (20.7%); Cuban (3.7%), and other (7.4%)  **Other demographics collected**: 71.1% completed the survey in English and 28.9% completed the survey in Spanish; 94.4% were born in the United States; 70.6% are from an urban environment; 23.6% in a suburban area, and 5.8% in rural areas | Referred to it as *"internalized homonegativity";* participants completed the Internalized Homonegativity Subscale of the Lesbian, Gay, and Bisexual Identity Scale, a 3-item subscale with responses ranging on a 6-point scale, from 1 (strongly disagree) to 6 (strongly agree); shown to be reliably and valid; a Spanish version was developed; strong reliability for this sample (α = 0.84) | Referred to as *“depression"*; participants completed the short version of the Center for Epidemiological Studies Depression Scale, a 10-item questionnaire on a 4-point rating scale ranging from 0 (rarely or none of the time) to 3 (most of the time); reliability was acceptable in a multiethnic/racial sample of adolescents; reliability in this sample was α = 0.73 | Results showed that internalized homonegativity was associated with depressive symptoms (β = 0.18, *p* < .05), with a positive correlation; as internalized homonegativity scores rose, so did depressive symptoms |
| Armelie, 2009^b^ | To examine relationships between self-criticism, internalized homophobia, and depressive symptoms | Quantitative cross-sectional study from a large Midwestern city in the United States using non-probability purposive sampling recruited from a queer community center | 65 self-identified sexual minority youth age in years range unknown; *M* = 19.22, *SD* = 2.11  **Sexual orientation:** 100% of youth identified as being on the queer spectrum, but the author did not provide demographic information  **Gender identity:** Male (*n* = 46, 70.8%); female (*n* = 19, 29.2%); does not specify if cisgender or transgender  **Racial or ethnic identity:**  Black/African American (*n* = 62, 95.4%); Hispanic/Latino/Chicano (*n* = 7, 10.8%); White (*n* = 4, 6.2%); Asian/Asian American (*n* = 2, 3.1%), Native American/American Indian (*n* = 6, 9.2%)  **Other demographics collected:** Authors also reported education level and employment status; 12th grade or GED (*n* = 29, 44.6%) and some college (*n* = 19, 29.2%); participants were mostly working full-time (*n* = 25, 38.5%), next largest subgroup was unemployed (*n*=18, 27.7%) | Referred to as *“internalized homophobia”*; participants completed The Sexual Identity Distress scale, a 7-items scale asking participants to indicate how much they agree or disagree on a 5-­point Likert scale with both positive and negative statements regarding their feelings about their sexual orientation; a total score was created by summing each item; the scale was found to have good internal reliability (α = 0.81) in the current sample | Referred to as “*depressive symptomatology”*; participants completed the Center for Epidemiological Studies Depression Scale for Children; to assess depression symptoms occurring within the past week, a 20-item measure adapted for use with  children and adolescents; the scale showed very good reliability in the present study (α = 0.916) | Results showed that internalized homophobia was associated and positively correlated with depressive symptoms (*r* = 0.274, *p* < .05); internalized homophobia predicted depressive symptoms independently (R^2^ = 0.075; β = 0.274; *t* = 2.261; *p* < .05), and when combined with self-criticism in a regression equation, the model significantly predicted depressive symptoms and accounted for a significant amount of variance (45%) |
| Bruce et al., 2014 *(including erratum)* | To explore minority stress on homelessness and health disparities among young men who have sex  with men | Quantitative cross-sectional study with a sample from the Chicago  Young Men’s Health Study, using non-probability, purposive and snowball sampling (e.g., online, community, and peer referral) | 200 assigned male at birth young men who have sex with men, ages 16-24 years; *M* = 20.88, *SD* = 2.09  **Sexual orientation:** Gay (*n* = 129, 64.5%); bisexual (*n* =57, 28.5%); other (*n* =14, 7.0%)  **Gender identity:** All identified as men  **Racial or ethnic identity:** African American (*n* = 76, 38.0%); Latina/o/e or Hispanic (*n* = 53, 26.5 %); White (*n* = 47, 23.5 %); multiracial/other (*n* = 18, 12%); Asian American (*n* = 4, 2.0%); Native American/Alaskan Native (*n* =2, 1.0%)  **Other demographics collected:** Authors also reported on experiences with homelessness, marijuana use, alcohol use, and other drug use | Referred to as “*internalization of sexual orientation stigma*”; participants completed a 9-item scale adapted from measures that were cited as previously validated in three studies prior to this article, with responses in four categories assessing agreement with different statements (1 was strongly disagree, 4 was strongly agree; α = 0.87) | Called “*depressive symptomology*”; participants completed the 20-item Center for Epidemiologic Studies depression scale (see Mereish et al., 2021 for description) | Results indicate that there were direct significant paths between internalization of sexual orientation stigma and major depressive symptoms within the past week (β = 0.89, *SE* = 0.25, OR = 2.44, 95% CI [1.63, 3.66], *p* < .001) |
| Bruce et al., 2015 | To explore resilience among  sexual minority male youth that may buffer the effects of  of minority stress | Same as Bruce et al., 2014 | Similar to Bruce et al., 2014, but here authors indicated 62% of individuals identified as gay as the final sample size was 194; they also did not report homelessness or substance use rates, but reported on educational level and employment with a majority of high school diploma and GED (*n* = 84, 42.0%); majority unemployed (*n* = 123, 61.5%) | Referred to as “*internalized homophobia*”; same as Bruce et al., 2014 | Referred to as “*major depressive symptoms*”; same as Bruce et al., 2014 | Results indicate that the final model demonstrated significant paths from internalized homophobia to major  depressive symptoms (β = 0.773, OR = 2.167, *p* <.001), with  internalized homophobia partially mediating the direct effect of  experience of sexual orientation stigma on major depression symptoms |
| Chodzen et al., 2019 | To examine the relationships between minority stress and mental health issues in transgender and gender-nonconforming youth and young adults | Quantitative cross-sectional study in a metropolitan Midwestern city in the United States, using non-probability, purposive, convenience sampling; from youth who completed screening questionnaires as standard care at their first clinical visit to an interdisciplinary gender program within a pediatric academic medical center | 109 transgender or gender nonconforming youth ages 12-28 years; *M* = 15.46, *SD* = 1.55  **Sexual orientation:** Not reported  **Gender identity:** Transgender (*n* = 94, 86.2%); gender nonconforming (*n* = 2, 1.8%); agender (*n* = 4, 3.7%); unsure/questioning (*n* = 5, 4.6%); on-binary (*n* = 3, 2.8%); genderqueer (*n* = 1, 0.9%)  **Racial or ethnic identity:** White (71.6%); Black or African American (1.8%); Hispanic American (8.3%); Asian American (2.8%); other (10.1%); 5.5% of missing data  **Other demographics collected:** Authors also reported sex assigned at birth; assigned female at birth (71.6%); assigned male at birth (28.4%) | Referred to as *“internalized transphobia”*, participants completed the Gender Minority Stress and Resilience measure; internalized transphobia was measured through a subscale where youth were asked the extent to which they agreed with each item on a 5-point Likert scale: 0 (strongly disagree), 1 (somewhat disagree), 2 (neither agree nor disagree), 3 (somewhat agree), and 4 (strongly agree); responses were summed and higher scores indicated higher levels of internalized transphobia | Referred to as diagnosis of “*Major Depressive Disorder (MDD)”;* participants completed the Youth Inventory (YI-4), a self-report scale used as an initial screening tool to evaluate DSM-IV criteria for several emotional and behavioral disorders among youth aged 12 to 18 years; youth reported the frequency of each symptom occurrence on a 4-point scale: 0 (never), 1 (sometimes), 2 (often), and 3 (very often); following the scoring guide, a sum of several endorsed symptoms indicated meeting diagnostic criteria | Results suggest that internalized transphobia predicts MDD; 33% of the youth (*n* = 36) met diagnostic criteria for MDD; for those that met MDD criteria, internalized transphobia odds ratios were 1.06; 95% CI from 1.00 to 1.12; *p* < .05; youth with higher scores of internalized transphobia were 1.06 times more likely to meet diagnostic criteria for MDD |
| Dyar et al., 2020 | To examine associations  between minority stressors, mental health, and substance use in sexual and gender minority individuals | Quantitative longitudinal study in the United States, using non-probability, purposive, convenience, and snowball sampling for sexual gender minority assigned female at birth; individuals from a previous cohort study were originally recruited in 2007; later cohorts were recruited on social media; researchers utilized 4 waves of data at 6 months between waves (6-month, 12-month, 18-month, and 24-month) for the 2016 cohort | 488 sexual and gender minority individuals assigned female at birth; *n* = 400 from the 2016 cohort; *n* = 88 from the 2007 cohort; ages 16-32 years; *M* = 20.06, *SD* = 3.66; demographics listed here are from Wave 1  **Sexual orientation:** Lesbian (*n* = 115, 23.6%); bisexual (*n* = 181, 37.1%); queer (*n* = 64, 13.1%); pansexual (*n* = 82, 16.8%); and other (*n* = 46, 9.4%)  **Gender identity:** Cisgender women (*n* = 360, 73.8%); transgender or male (*n* = 44, 9%), and genderqueer/nonbinary (*n* = 84, 17.2%)  **Racial or ethnic identity:** White (*n* = 127, 26%); Black (*n* = 170, 34.8%), Latina/o/e (*n* = 120, 24.6%), and other (*n* = 71, 14.5%)  **Other demographics collected:** Authors also reported household income, with the largest categories being $20,000-$39,999 (*n* = 110, 22.5%) and >$80,000 (*n* = 22.3%) | Referred to as *“sexual orientation-based internalized stigma”*; participants completed an 8-item desire to be heterosexual subscale (from Puckett et al., 2017); participants were asked how much they agreed with statements on a scale of 1 (strongly disagree) to 4 (strongly agree); items were averaged to create a score (α = .84 –.89) | Referred to as *“depression”;* participants completed the Patient-Reported Outcomes Measurement Information System (PROMIS) Depression Short Forms 8a; scales include 8-items measured on a scale of 1 (never) to 5 (always); there was strong internal consistency and content validity (α = 0.95) | Results show that internalized stigma was positively associated with depression (*r* = .13, *p* < .05); within-person internalized stigma was also prospectively associated with depression 6 months later (β=.16 [95% CI .08, .23]); for between-person associations in general, individuals who experienced more internalized stigma on average across time points generally experienced more depressive symptoms across time points; within, concurrent (β = .08 [95% CI .05, .11]) and between, concurrent (β=.34 [95% CI .03, .75)] |
| Dyar et al., 2021 | To examine the associations between stressors unique to bisexual+ individuals and internalizing symptoms | The design, data, location, and sampling are similar to Dyar et al., 2020; however, they analyzed 5 waves of primary data (Wave 2 to Wave 6, at 30 months) | 414 bisexual+ individuals (attracted to more than one gender) assigned female at birth *n* = 354 for the 2016 cohort; *n* = 60 for the 2007 cohort (FAB 400 cohort, see Dyar et al., 2020); ages 16-32 years; *M* = 20.21, *SD* = 3.37; demographics listed here are from Wave 2  **Sexual orientation:** Bisexual (*n* = 175, 42.3%); queer (*n* = 77, 18.6%); pansexual (*n* = 74, 17.9%); other (*n* = 88, 21.3%)  **Gender identity:** Cisgender women (*n* = 294, 71%); transgender or male (*n* = 38, 9.2%); and genderqueer or nonbinary (*n* = 82, 19.8%)  **Racial or ethnic identity:**  White (*n* = 112, 27.1%); Black (*n* = 136, 32.9%); Latina/o/e (*n* = 104, 25.1%); and other (*n* = 62, 15%)  **Other demographics collected:** Authors also reported highest education (the majority with some college, *n*= 208, 50.2%) and household income (most were >$80,000, *n*= 103, 24.9%) | Referred to as *“internalized bi+ stigma”,* participants completed a survey with subscales of the Bisexual Identity Inventory, a 5-item subscale; participants respond to items on a scale of 1 (strongly disagree) to 7 (strongly agree) and items were averaged; acceptable internal consistency (α = 0.70 – 0.85). | Same as Dyar et al., 2020 | Results indicate that internalized bi+ stigma is positively correlated with depression (within-person *r* = 0.13, *p* < .01; between-persons *r* = 0.25, *p* < .01); at the within-person level internalized bi+ stigma was associated with depression symptoms; as bi+ stigma increased, so did depressive symptoms over the next 6 months (*b* = 0.71, 95% CI .19, 1.23, *p* = .01); between-persons (*b* = 2.48, 95% CI 1.15, 3.95, p < .001) indicated internalized bi+ stigma was associated with depressive symptoms; people who had higher levels of stigma across all study waves tended to experience more symptoms of depression |
| Goldbach et al., 2015 | To explore minority stress and its relationship to substance use, suicide, and depression among lesbian, gay, and bisexual adolescents | Same as Gibbs & Goldbach, 2015 | 1,911 queer youth ages 12-17 years; *M* = 15.79, *SD* = 1.2 years  White n=1,524 (80%), otherwise labelled as racial or ethnic minority n=387 (20%); residential status included as live with parent (n=1,758 or 92%)  **Sexual orientation:** Gay (*n* = 1,044, 55%); lesbian (*n* = 207, 11%); bisexual (*n* = 660, 35%)  **Gender identity:** Not reported  **Racial or ethnic identity:** White (*n* = 1,524, 80%); otherwise labelled as racial or ethnic minority (*n* = 387, 20%)  **Other demographics collected:** Authors reported the percentage of youth using marijuana (*n* = 1,524, 80%), and their residential status included as living with family (*n* = 1,758, 92%) | Same as Gibbs & Goldbach, 2015 | Referred to as *“psychological distress*”, participants answered one binary depression-related item: “Have you ever felt hopeless due to being queer?”; researchers also measured suicide risk and combined this into their analysis. Participants answered two binary suicide risk-related items: “Have you ever tried to harm yourself due to being queer?”, and, “Have you ever attempted suicide due to being queer?” | Results show that internalized homophobia was positively related to psychological distress (β = 0.263, *t* = 6.403, *p* < .001); just over half of participants felt hopeless in the last 30 days (*n* = 966, 51%); reported confirmatory factor analysis for negative feelings about one’s sexual orientation and their desire to change their sexual orientation within the internalized homophobia factor (*r* = 0.483; *p* < .001). Regarding suicide risk-related items, *n* = 551 (29%) attempted to harm themselves, and *n* = 436 (23%) attempted suicide. |
| Gutiérrez, 2021^b^ | To explore the relationships of parent support, religion, and sexuality  conflict, internalized homophobia, and self-compassion on depressive symptoms among queer young adults | Quantitative cross-sectional survey data and correlational study, using a combination of non-probability purposive, convenience, and snowball sampling; the survey was available online for individuals to access across the United States | 235 cisgender queer individuals ages 18-25 years; *M* = 21.31, *SD* = 2.27 years  **Sexual orientation:** Bisexual (*n* = 113, 48.1%); gay (*n* = 45, 19.1%); lesbian (*n* = 31, 13.2%); pansexual (*n* = 19, 8.1%); queer (*n* = 9, 3.8%); asexual (*n* = 9; 3.8%); questioning (*n* = 4; 1.7%); other (*n* = 5, 2.1%);  **Gender identity:** Men (*n* = 113, 48.1%); women (*n* = 107, 45.5%); nonbinary (*n* = 5, 2.1%); genderqueer (*n* = 3, 1.3%); nonconforming (*n* = 5, 2.1%); other (*n* = 2, 0.9%)  **Racial or ethnic identity:** White (*n* = 181, 77%); African American or Black (*n*=16, 6.8%); Asian or Asian-American (*n* = 20, 8.5%); Hispanic or Latina/o/e (*n* = 35, 14.9%); Middle Eastern (*n* = 3, 1.3%); Native Hawaiian or other Pacific Islander (*n* = 1, 0.4%); other (*n* = 5, 2.1%)  **Other demographics collected:** The author also collected data on religious affiliation (Agnostic or Athiest, 62.5%); marital status (the largest group was single and never married, 87.7%); parent sexual orientation (majority of two heterosexual biological parents, 80.4%); education levels (majority some college credit with no degree, 36.6%; Bachelor’s degree, 25.1%); gender of current partner, and occupation | Referred to as “*internalized homonegativity*”, participants answered items from the adapted version of the Lesbian, Gay, and Bisexual Identity Scale, a 27-item scale with moderate to high levels of internal consistency and reliability (α =.72 to .94); internalized homonegativity is 1 of 8 subscales, rated using a six-point Likert scale (from strongly disagree to strongly agree) | Identified as *“depression”,* participants completed the Patient Health Questionnaire, a 9-item screening tool used by clinicians to diagnose depressive disorders and based on DSM-IV criteria; respondents indicate how often during the previous two weeks, they were bothered by symptoms or problems, rated on a 4-point Likert-type scale ranging from 0 (not at all) to 3 (nearly every day), with higher scores indicating more symptoms and higher severity; in previous studies α =.86 to .89 | Results indicate a lack of normality (positive skewness, low levels in sample) in regard to internalized homonegativity; in analyzing the full path model, the correlation between internalized homophobia and higher levels of depression was not significant (*r* = 0.071, *p* = .278, *p* > .05) |
| Jackman et al., 2018 | To examine internalized transnegativity and psychological distress in two age groups of transgender individuals | Quantitative cross-sectional survey study in the United States with non-probability, convenience, purposive sampling; this sample was drawn from a larger study and consists of secondary data analysis comparing younger and older transfeminine cohorts in an online survey | 133 transgender individuals who identify as being on the feminine spectrum despite being assigned male at birth ("trans-feminine individuals") from the younger cohort, ages 18-24 years; *M* = 21.17, *SD* = 1.99 years  **Sexual orientation:** Not explicitly reported by name; primarily attracted to men (37.9%); primarily attracted to women (21.2%), attracted to both men and women (40.9%)  **Gender identity:** Transsexual (37.9%); crossdresser (16.7%); drag queen or female impersonator (15.9%); other transgender identity (e.g., bigender, genderqueer, two-spirit, 29.5%)  **Racial or ethnic identity** White (*n* = 99, 74.4%); other racial or ethnic identities not reported  **Other demographics collected:** Researchers also collected information on education levels, majority reported at least some college (78.5%); median annual household income was $17,500 | Referred to as “*internalized transnegativity*”; participants completed a 26-item Transgender Identity Survey that assesses internalized transnegativity among different dimensions like pride, shame, and alienation from transgender peers; response options were on a 7-point Likert scale ranging from 1 (strongly disagree) to 7 (strongly agree); total scores were the average of all 26 items; high internal consistency and reliability (α = 0.94) | Referred to as *“psychological distress”*; participants completed the Brief Symptom Inventory 18, an 18-item scale that assesses depression, anxiety, and somatization; participants were asked how much a particular symptom distressed or bothered them during the last 7 days on a Likert-scale (1 = not at all to 5 = extreme); scores were added up using the Global Severity Index, and higher scores indicated more psychological distress (α = 0.94) | Results indicate that younger participants reported higher levels of internalized transnegativity (*p* < .05), and higher levels of psychological distress (*p* < .001) than the older cohort; when looking at the entire sample (*N* = 440), the relationship between internalized transnegativity and psychological distress was significant (*b* = 3.72, *SE* = 0.71, *p* < .001). Authors contacted researchers who used a simple regression predicting psychological distress with the independent variable internalized transnegativity, β = 0.30 |
| Katz-Wise et al., 2021 | To examine the longitudinal effects of gender minority stressors on substance use in gender minority adolescents, and related risk factors (e.g., internalized transphobia, depressive symptoms) and protective factors | Longitudinal quantitative survey study with 5 waves of data collection from the United States community-based Trans Teen and Family Narratives Project through Boston Children’s Hospital; wave data was collected through an online survey every 6 months across 2 years; surveys were collected following a qualitative interview from a larger mixed-methods study; sampling techniques were not discussed | 30 gender minority youth from the New England area, ages 13-17 years; *M* = 15.1, *SD* = 1.1 years  **Sexual orientation**: Completely straight/heterosexual (*n* = 6, 20%); mostly straight/heterosexual (*n* = 2, 6.7%); bisexual (*n* = 5, 16.7%); mostly lesbian/gay (*n* = 5, 16.7%); completely lesbian/gay (*n* = 4, 13.3%); queer (*n* = 7, 23.3%); pansexual (*n* = 13, 43.3%); questioning (*n* = 2, 6.7%)  **Gender identity:** Transfeminine (*n* = 11, 36.7%); transmasculine (*n* = 15, 50%); non-binary (*n* = 4, 13.3%)  **Racial or ethnic identity:** White (*n* = 22, 73.3%); Hispanic or Latina/o/e (*n* = 4, 13.3%); Asian, Native Hawaiian or other Pacific Islander (*n* = 2, 6.7%); American Indian or Alaska Native (*n* = 1, 3.3%); mixed race/ethnicity (*n* = 1, 3.3%)  **Other demographics collected:** Assigned sex at birth demographics were collected; assigned female at birth (*n* = 18, 60%); assigned male at birth (*n* = 12, 40%) | Referred to as “*internalized transphobia*”, participants in waves 1-5 completed the Gender Minority Stress and Resilience Measure's 8-item subscale; response options were on a 5-point Likert scale from 0 (strongly disagree) to 4 (strongly agree); scores were summed item responses ranging from 0–24; higher scores indicated greater internalized transphobia (α = 0.90) | Called *“depression”,* respondents completed a survey for the past week using the 10-item Center for Epidemiologic Studies Depression Scale–Short Form, with answers on a Likert-type scale from 0 (rarely or none of the time–less than 1 day) to 3 (most or all of the time– 5–7 days); items worded in the opposite valence were reverse-coded and a scale score was created by summing item responses with a range of 0–30; higher scores indicated greater depressive symptoms(α = 0.88) | Results show associations between internalized transphobia and depressive symptoms (*r* = 0.37, *p* < .001); internalized transphobia was associated with increased odds of depressive symptoms over time; internalized transphobia to depressive and anxious symptoms OR (95% CI)= 1.41 (1.20, 1.66) |
| Langdon, 2010^b^ | To explore the influence of spiritual coping in the lives of African American queer adolescents and determine if psychological well-being is predicted by internalized homophobia | Quantitative cross-sectional survey study from the mid-Atlantic area in the United States using non-probability, purposive sampling techniques through a two-prong approach from community agencies  (e.g., counseling, support groups, education), and from undergraduate instructors and campus organizations  at historically Black colleges and universities | 86 individuals from the mid-Atlantic area who self-identified as African American and as gay, lesbian, or bisexual, ages 18-23 years; *M* = 19.57, *SD* = 1.45 years  **Sexual orientation:** Lesbian or gay (*n* = 58, 67.5%); bisexual (*n* = 26, 30.2%)  **Gender identity:** Male (*n* = 51, 59.3%); female (*n* = 35, 40.7%); the author placed transgender identity (*n* = 2, 3.2%) under “sexual orientation”  **Racial or ethnic identity:** 100% of respondents self-identified as African American  **Other demographics collected:** Authors also reported education, sexual orientation, out to family, if respondents bring significant others to family events, adolescent community, current community, religious affiliation, how regularly they attend a religious service, religious activities, how often they read religious materials, commitment to faith, distinction between religiosity and spirituality, and how often they pray; the majority of participants had completed high school (*n* = 62, 87.3%); identified as being “out” to their family (*n* = 61, 71.8%); were Christian (*n* = 59, 69.4%,); and grew up or currently reside in suburban or urban environments (90%) | Referred to as *“internalized homophobia/homonegativity”;* respondents completed the Revised Homosexuality Attitude Inventory, a 21-item self-report inventory that assesses internalized homophobia or negative views of one's sexual orientation on a 4-point Likert-type scale from 1 (strongly disagree) through 4 (strongly agree), with high scores indicating more internalized  homonegativity; two scores can be derived (personal and global homonegativity; α = 0.82 and 0.76 respectively) | Referred to as *“psychological distress”*; participants completed the Brief Symptom Inventory 18 (refer to Jackman et al., 2018 for description); the author reported that the items assessed were three symptom domains (somatization, depression, and Anxiety), and a Global Severity Index | Results indicate that neither personal homonegativity (*r* = 0.214) nor global homonegativity (*r* = .021) were significantly correlated with the Brief Symptom Inventory psychological distress; internalized homophobia did not predict significant variance in the Brief Symptom Inventory for psychological distress, scores (R^2^ = .199, *F*[9, 50] = 1.38, *p* = .22, *b* = 0.375, *SE* = 0.307, β = 0.217) |
| Mereish et al., 2021 | To examine minority stressors over a 21-day monitoring period and examine how these experiences relate to same-day affect among queer adolescents | Longitudinal quantitative cohort study in a Mid-Atlantic metropolitan city; individuals were recruited through non-probability, purposive sampling techniques through school and community-based organizations and queer student clubs | 94 queer youth ages 12-18 years; *M* = 16.1, *SD* = 1.5 years  **Sexual orientation:** Bisexual (35.1%); lesbian (17%); pansexual (16%); gay (12.8%); queer (10.6%); asexual (5.3%); questioning (2.1%), and don’t know (1.1%)  **Gender identity:** Female (58.5%); male (10.6%); gender queer/gender nonconforming (11.7%); transgender male/boy/man (11.7%); or a different gender identity (e.g., agender, gender fluid, 7.4%); 68.1% of the participants were cisgender (*n* = 64) and 31.9% were a gender minority (*n* = 30)  **Racial or ethnic identity:** White (54.8%); Black/African American (23.7%); Hispanic/Latina/o/e (11.7%); biracial or multiracial (16.1%); Asian/Asian American or Pacific Islander (2.2%); and other (3.2%)  **Other demographics collected:** Researchers also collected information on education level, and status on free and reduced lunches at school; majority in high school (81.9%); middle school (8.5%); other incoming first-year or first-year student in college (9.6%); 34% received free or reduced lunch at school | Referred to as “*internalized homonegativity*”; participants completed part of the Sexual Minority Adolescent Stress Inventory Scale, which measures lifetime experiences of sexual minority stress and consists of 54-items which assess 10 dimensions of sexual minority stress, including internalized homonegativity; acceptable reliability in this sample (α = 0.95); to assess one form of proximal minority stress, they used the seven-item Internalized Homonegativity subscale with binary responses (α = 0.79) | Called *“depressive symptoms”*; youth completed the Center of Epidemiologic Studies Depression Scale and rated 20 items on a 4-point Likert-type scale from 0 (rarely or none of the time, less than 1 day) to 3 (most or all of the time, 5–7 days); this scale is a widely used, valid, and reliable scale of depression in youth and adults; demonstrated strong internal consistency in the present study (α = 0.88) | Results show a positive correlation between internalized homonegativity and baselines depressive symptoms (*r* = 0.21, *p* *<* .01) |
| Puckett et al., 2017^a^ | To examine the role of minority stressors and intrapersonal factors in predicting suicidality and depression in queer youth from the Mid-South | A quantitative, cross-sectional cohort study using non-probability, purposive sampling, and snowball sampling techniques by recruiting youth through queer community organizations and word of mouth at high schools in the United States (Memphis, Tennessee) | 61 lesbian, gay, and bisexual youth in the Memphis, Tennessee area ages 14-23 years; *M* = 17.98, *SD* = 1.93  **Sexual orientation:** "Totally" gay or lesbian (*n* = 29, 47.5%); "almost totally" gay or lesbian (*n* = 11, 18%); "mostly" gay or lesbian (*n* = 3, 4.9%); equally gay or heterosexual (*n* = 1, 1.6%); bisexual equally attracted to both sexes (*n* = 4, 6.6%); bisexual more attracted to same sex (*n* = 5, 8.2%); bisexual more attracted to other sex (*n* = 4, 6.6%); queer (*n* = 2, 3.3%); bi-curious (*n* = 1, 1.6%); other (*n* = 1, 1.6%)  **Gender identity:** Female (*n* = 38, 62.3%); male (*n* = 23, 37.7%)  **Racial or ethnic identity:** White (*n* = 45, 73.8%); Black/African American (*n* = 9, 14.8%); Asian American (*n* = 2, 3.3%); Native American (*n* = 1, 1.6%); Indian (*n* = 1, 1.6%); Cuban (*n* = 1, 1.6%); biracial/multiracial (*n* = 1, 1.6%); other (*n* = 1, 1.6%)  **Other demographics collected:** Researchers partially reported on parent education, with most participants from families with parents who had received some higher education beyond high school or with a higher degree | Referred to as *“internalized heterosexism”*; youth completed the 20-item Revised Homosexuality Attitudes Inventory; items were measured on a 4-point scale (1 is strongly disagree, 4 is strongly agree); responses were summed to create a total score, with higher scores indicating higher levels of internalized heterosexism (α = 0.84) | Referred to as “*depression*”, participants completed the 20-item Center for Epidemiological Studies in Depression Scale (see Mereish et al., 2021 for description); in this study α = 0.90 | Results indicate that internalized heterosexism was correlated with depression (*r* = 0.31, *p* < .05); internalized heterosexism was significant in predicting depression scores (*b* = 0.72, *SE* = 0.31, β = 0.32, *p* < .05) |
| Sarno et al., 2020 | To examine relationships between rumination, minority stressors, and depressive symptoms in a sample of young men who have sex with men and young transgender women | Longitudinal quantitative study with individuals from the Chicago metropolitan area of the United States; data were taken from baseline, 6-month, and 1-year assessments from a large cohort; this data was taken from a larger study called RADAR with young men who have sex with men, using non-probability, purposive, and snowball sampling sampling from two cohort studies who were first recruited in 2007 | 1,130 young men who have sex with men and young transgender women from the Chicago metropolitan area, ages 16-29 years; *M* = 21.38, *SD* = 3.02  **Sexual orientation:** Gay (69.8%); bisexual (20.9%); queer (3.3%); straight/heterosexual (1.7%); unsure/questioning (1.5%); lesbian (0.1%); another orientation not listed (2.7%)  **Gender identity:** Male (92.1%); transgender male-to-female (5.2%); or another identity not listed (2.7%)  **Racial or ethnic identity:** Black or African American (33.6%); Hispanic/Latina/o/e (30.2%); White (25.1%); multiracial (7.8%); Asian (2.4%); American Indian or Alaska Native (0.2%); Native Hawaiian or other Pacific Islander (0.2%); another identity not listed (0.6%)  **Other demographics collected:** Authors did not specify any other demographic information | Referred to as “*internalized stigma*”; participants completed 8 items from a 22-item adapted and validated scale (per Puckett et al., 2017^c^); individuals completed the Desire to Be Straight subscale, whereby each item is rated on a 4-point Likert-type scale ranging from 1 (strongly disagree) to 4 (strongly agree); items are averaged into a single score, with a higher score indicating a higher level of internalized stigma (baseline α = 0.89). | Identified as “*depression*”; participants completed the Patient-Reported Outcomes Measurement Information System (PROMIS) Depression Short Form 8a (see Dyar et al., 2020 for description); the internal reliability of this  measure was high at each time point (α = 0.95) | Results of the bivariate correlations indicate that internalized stigma was statistically significantly positively correlated with depression at all three time periods (e.g., baseline, *r* = 0.25, *p* < .01); the models indicated mixed results as the main effects of internalized stigma at Time 1 on depression at Time 3, controlling for depression at Time 1, were not significant (β = .02, *SE* =0.03, *p* = .43); however, indirect effects of internalized stigma at time 1 on depression at time 3 were significant (β = 0.01, *p* = .014); internalized stigma at Time 1 significantly predicted increased rumination at Time 2 (β = 0.13, *p* < .001), which, in turn, significantly predicted higher levels of depression at Time 3 (β = 0.11, *p* < .05) |
| Simonson, 2012^b^ | To examine the relationships between general and sexual minority-specific stress, vulnerabilities, and depression symptoms in queer youth | A longitudinal quantitative prospective study with two assessments 6 weeks apart using non-probability, purposive and snowball sampling techniques to recruit queer youth in the United States (Seattle, Washington area) from queer youth centers and schools | 135 queer youth in the Seattle, Washington area were retained for both assessments (whereas the overall *N* = 165), ages 13-22 years; *M* = 18.86, *SD* = 2.05  **Sexual orientation**: Exclusively gay/lesbian (*n* = 64, 38.8%); mostly gay/lesbian (*n* = 34, 20.6%); bisexual leaning gay/lesbian (*n* = 18, 10.9%); bisexual (*n* = 29, 17.6%); bisexual leaning straight (*n* = 9, 5.5%); mostly straight (*n* = 10, 6.1%); exclusively straight (none)  **Gender identity:** Feminine (*n* = 78, 47.3); masculine (*n* = 46, 27.9%); queer (*n* = 26, 15.8%); transgender (*n* =8, 4.8%); other (*n* =7, 4.2%)  **Racial or ethnic identity:** White (81.8%); African American (7.5%); Asian (5.5%); Native American/Alaska Native (4.8%); Native Hawaiian/Pacific Islander (1.8%); another race (7.9%); author reports approximately 9.3% of the sample selected more than one racial category; ethnically, 7.3% of the sample identified themselves as Hispanic  **Other demographics collected:** Author also reported biological sex as male (*n* =58, 35.2%); female (*n* = 105, 63.6%); other (*n* =2, 1.2%) | Referred to as “*internalized homophobia*”, participants completed the Short Internalized Homonegativity Scale, a 12-item self-report measure where respondents endorse the extent to which they agree with each statement on a 7-point Likert-type scale ranging from 1 for strongly disagree to 7 for strongly agree; higher scores indicate higher internalized homonegativity; previously reported reliability of α = 0.78 | Referred to as “*depressive symptoms*”; participants completed the Center for Epidemiological Studies Depression scale (see Mereish et al., 2021) | Results showed that internalized homophobia was significantly correlated with depressive symptoms in part 1 (*r* = 0.19, *p* < .05), but not in part 2; in models, multiple steps showed that internalized homophobia became a moderately significant predictor of depressive symptoms in the combined model (*b* = 0.23 [*SE* = 0.08], *p* < .001) |
| Swann et al., 2019 | To explore associations between victimization, mental health, and substance use  among young men who have sex with men, and how associations change  over time | Longitudinal quantitative cohort study (surveyed every 6 months for 2.5 years); six waves of data were included in these analyses, and participants were recruited through non-probability convenience sampling followed by a modified form of respondent-driven sampling; data is part of Crew 450, an ongoing longitudinal cohort study of young men who have sex with men in the United States Chicago metropolitan area | 450 young men who have sex with men in the Chicago metropolitan area, baseline ages 16-20 years; *M* = 18.93, *SD* = 1.29  **Sexual orientation:** Only gay/homosexual (*n* = 226, 50.2%); mostly gay/homosexual (*n* = 103, 22.9%); bisexual (*n* = 96, 21.3%); mostly heterosexual (*n* = 11, 2.4%); other (*n* =11, 2.4%); only heterosexual (*n* = 3, 0.7%)  **Gender identity:** Authors did not report, but all individuals in the study identified as men who have sex with men  **Racial or ethnic identity:** African American/Black (*n* = 240, 53.3%); Hispanic/Latina/o/e (*n* = 90, 20.0%); non-Hispanic White (*n* = 81, 18.0%); participants who identified their race as  other (*n* = 24, 5.3%); Asian (*n* = 8, 1.8%); American Indian or Alaska Native (*n* = 7, 1.6%)  **Other demographics collected:**  Authors reported retention rates over the six waves of data with follow-up waves reporting 85.8%, 80.7%, 75.6%, 75.4%, and 75.6% respectively; highest level of education the majority was some high school (*n* = 172, 38.2%), followed by  some college (*n* = 152, 33.8%);  most identified as current students (75.1%) and were not currently working (68.9%) | Referred to as “*internalized homophobia*”, respondents completed an 8-item desire to be straight subscale, which comes from The Internalized Gay-Related Stigma measure created for this study; all items in this subscale were originally used in the Internalized Homosexual Stigma scale and  validated for this sample; items are scored on a 4-point scale (1 is strongly disagree, 4 is strongly agree), with higher scores indicating more internalized homophobia; the subscale was computed by taking the mean of all items, and reliability at baseline was good (α = 0.88) | Called “*internalizing problems*”; participants completed the Adult Self-Report developed by the Achenbach System of Empirically Based Assessment, which uses a 3-point response scale where 0 is not true, and 2 is very true or often true; total scores for eight syndrome scales were calculated by taking the sum of the Achenbach System of Empirically Based Assessment–identified items; syndrome scales Anxious/Depressed, Withdrawn, and Somatic Complaints created this score (α = 0.93) | Results indicated that higher internalized stigma was significantly associated with higher internalizing problems (*b* = 2.55, *p* < .001, 95% CI -1.75, 3.45) |
| Walker & Longmire-Avital, 2013 | To examine the relationships between religious faith, internalized homonegativity, and resiliency for Black  lesbian, gay, and bisexual emerging adults | Quantitative cross-sectional study using non-probability, purposive sampling through online recruitment methods (e.g., listservs through the Black Student Unions, LGBT centers, and religious organizations) | 175 Black young adults who self-identify as gay, lesbian or bisexual; individuals were recruited from multiple areas throughout the United States, as surveys were available online and therefore adults across the United States could access them; ages 18-25 years; *M* = 21.34, *SD* = 2.36  **Sexual orientation:** Gay (*n* = 66, 67%); lesbian (*n* = 50, 66%); bisexual (*n* = 33, 33.3%)  **Gender identity:** Male (*n* = 99, 56.6%); female (*n* = 76, 43.4%)  **Racial or ethnic identity:** All individuals self-identified as Black  **Other demographics collected:** Researchers also collected data on education, annual family income, religious/spiritual identity, and current religion; the majority of participants were college students (*n* = 51, 59.3%) with an annual family income of $20,000 to $49,999 (*n* = 31, 36%), most identified as spiritual (*n* = 42, 49.4%) and selected Christianity as their religion (*n* = 43, 50.6%) | Referred to as *“internalized homonegativity*”; respondents completed a 23-item inventory, Mayfield’s 2001 Internalized Homonegativity Inventory that measures internalized negative feelings toward homosexuality; responses are on a 6-point Likert scale and range from 1 to 6 (strongly disagree to strongly agree; α = 0.93) | Called *“depression”*, participants completed the 20-item Center for Epidemiological Studies Depression scale (α = 0.92; see Mereish et al., 2021 for description) | Results of the bivariate analyses show a positive correlation between internalized homonegativity and depression (*r* = 0.16, *p* < .05); levels of homonegativity varied depending on gender identity and sexual orientation (e.g., bisexual men reported higher levels of internalized homonegativity than bisexual women, gay men, or lesbian women in this study); bisexual women reported higher levels of depression than gay men |
| Zhao et al., 2021 | To examine relationships between minority stress and depressive symptoms among queer Latina/o /e youth | Quantitative cross-sectional cohort study with a smaller analytic sample drawn from a larger study (see Anhalt et al., 2020) through convenience, purposive, non-probability sampling designs online via Gay, Lesbian, and Straight Education Network (GLSEN) social media accounts | 238 Latina/o/e queer youth from multiple areas throughout the United States ages 14-24 years; *M* = 18.98, *SD* = 2.31  **Sexual orientation:** Gay (54.2%); lesbian (18.9%); bisexual (8%); queer (9.7%); questioning (12.2%); pansexual (6.3%); asexual (0.8%); researchers allowed participants to check all that apply  **Gender identity:** Male (69.3%); female (21.8%); transgender (2.1%); nonbinary (5%); does not apply (2.1%)  **Racial or ethnic identity:** All individuals self-identified as Latina/o/e  **Other demographics collected:** Researchers also collected data on socioeconomic status, with the largest categories being $30,000 to $39,999, $40,000 to $49,999, and $50,000 to $74,999 (all at 22.1%); youth that attended high school (*n* = 112 and college (*n* = 126) | Referred to as “*internalized homonegativity*”; respondents completed a 3-item revised Private Collective Self-Esteem Scale, and items were rated on a 6-point Likert-type scale (1 was strongly disagree; 6 was strongly agree); the mean was used for analysis, with higher scores reflecting higher levels of internalized homonegativity (α = 0.85) | Referred to as “*depressive symptoms*”; participants completed the 10-item short version of the Center for Epidemiological Studies Depression Scale (see Anhalt et al., 2020 for description); current sample α = 0.75 | Results show a correlation between internalized homonegativity and depressive symptoms (*r* = 0.39, *p* < .01); internalized homonegativity was significantly associated with depressive symptoms (*b* =0.07, *p* < .05). However, the association between internalized homonegativity and depressive symptoms is not significantly different between high school and college, and the standardized coefficient for this path in the final model is β = 0.17. |
| ^a^ Indicates this study examined both depressive and suicide risk outcomes, and is the same study cited in Appendix E (*n* = 1)  ^b^ Indicates grey literature (i.e., master’s thesis or doctoral dissertation) | | | | | | |

**Appendix E**

*Summary of Studies Included in the Systematic Review Related to Suicide Risk Outcomes (n = 4)*

| Citation | Study Purpose | Study Design, Location and Sampling Strategy | Sample Description | Measurement of Internalized Stigma | Measurement of Suicide Risk | Results and Findings on the Relationship(s) Between Internalized Stigma and Suicide Risk |
| --- | --- | --- | --- | --- | --- | --- |
| Austin et al., 2020 | To better understand suicide risk among transgender youth across the United States and  Canada | Quantitative cross-sectional cohort study and online survey with data collected from March to July 2016; data was pulled from an online study called Project #Queery, of sexual and gender minority youth across the United States and Canada using non-probability purposive sampling through a multipronged, targeted approach to recruit a diverse sample | 372 youth ages 14-18 years; *M =* 15.99*, SD =* 1.232; 281 (75.5%) youth were from the United States, 89 (23.9%) youth were from Canada, and 2 from “other”; demographics as outlined below are non-mutually exclusive  **Sexual orientation:** Gay (*n* = 49, 13.2%); lesbian (*n* = 15, 4.0%); bisexual umbrella (*n* = 60, 16.1%); queer (*n* = 93, 25%); questioning (*n* = 39, 10.5%); pansexual umbrella (*n* = 160, 43%); asexual umbrella (*n* = 68, 18.3%); other (*n* = 8, 2.2%); two-spirit (*n* = 3, 1.8%); straight (*n* = 18, 4.3%)  **Gender identity:** Transgender man (*n* = 332, 89.2%); transgender woman (*n* = 35, 11.6%); nonbinary/gender fluid (*n* = 122, 32.8%); transgender (*n* =1, 0.3%); man (*n* = 4, 9.4%); woman (*n* = 12, 3.2%); demiboy (*n* = 4, 1.1%); agender (*n* = 4, 1.1%); two-spirit (*n* =2 , 0.5%); other (*n* = 3, 0.8%)  **Racial or ethnic identity:** Black (*n* = 15, 4.0%); American Indian/Indigenous (*n* = 21, 5.6%); White (*n* = 308, 82.8%), Hispanic (*n* = 33, 8.9%); Asian (*n* = 13, 3.5%); mixed race (*n* = 27, 7.3%)  **Other demographics collected:** Researchers also reported on type of community; 45.2% (*n* = 154) of individuals were from towns of 2,500-50,000 people, 36.1% (*n* = 123) were from a city of more than 50,000 people, and 17.3% (*n* = 59) of youth were from rural areas of less than 2,500 people | Referred to as *“internalized self- stigma”*; participants completed a 5-item scale to assess their own personal (self) internalized lesbian, gay, bisexual, transgender, queer-related stigma; the scale was adapted from the Nungesser Homosexual Attitudes Inventory (NHAI, Nungesser, 1983); the 5-item responses utilized a 5-point response scale ranging from strongly disagree to strongly agree; current sample α = 0.73 | Suicide risk was assessed through *“lifetime suicide attempt”* and *“past 6-month suicidality*”; *suicidality* was measured using 2 single-item variables from the Diagnostic and Statistical Manual of Mental Disorders 5^th^ Edition; the first item explored suicidal ideation using a 4-point Likert-type scale (e.g., never, hardly ever, sometimes, often) and the question, “In the past 6 months I thought about killing myself or committing suicide.” Past 6-month suicidality was recoded into a dichotomous yes/no variable, and lifetime suicide attempts were assessed using the following dichotomous question, “Have you ever tried to commit suicide?” | Analyses were conducted to explore the impact of internalized self-stigma on both past 6-month suicidality and lifetime suicide attempts; results show significant associations (*p* < .01) between internalized self-stigma and both lifetime suicide attempts and past 6-month suicidality. In the full model with all independent variables, internalized self-stigma (OR = 1.2, *p* < .05) made a unique, statistically significant contribution to the model of past 6-month suicidality (*B* = 0.136, *SE* = 0.057). However, in the full model examining all correlates of lifetime suicide attempts, internalized stigma did not make a unique, statistically significant contribution. |
| Gibbs & Goldbach, 2015 | To examine if religious and queer identity  conflict indicators are associated with  suicidality, and if internalized homophobia mediates this relationship | Quantitative cross-sectional survey, secondary data analysis using a national dataset collected by OutProud in 2000; non-probability purposive sampling through survey links made available through online and in-print outlets, and websites  that cater to young queer persons throughout the United States | 2,949 queer young adults ages 18-24 years; *M* = 20.07, *SD* = 1.98  **Sexual orientation:** Gay/lesbian (*n* = 1,814, 61.5%; bisexual (*n* = 788, 26.7%); heterosexual (*n* = 5, 0.2%), questioning (*n* = 285, 9.7%), and other (*n* = 57, 1.9%)  **Gender identity:** Cisgender male (*n* = 2,230, 75.6%); cisgender female (*n* = 644, 21.8%); transgender male (*n* = 21, 0.7%); transgender female (*n* = 27, 0.9%); other (*n* = 27, 0.9%)  **Racial or ethnic identity:** Black or African American (*n* = 84, 2.8%), White (*n* = 2,420, 82.1%), Latina/o/e (*n* = 13, 4.4%); Asian (*n* = 126, 4.3%); other (*n* = 189, 6.4%)  **Other demographics collected:** Authors also included information on religious affiliation, with a majority Christian Protestant (*n* = 549, 18.7%); Christian Catholic (*n* = 422, 14.4%); and non-religious (*n* = 733, 25%) | Referred to as *“internalized homophobia”*, a total score ranging from 0 to 3 was created by adding up three dichotomous items answered by participants, to assess comfort with being queer, desire to not be queer, and desire to change from being queer; dichotomous items were then totaled to equal the total of internalized homophobia, with higher values indicating more internalized homophobia | Suicide risk was measured through three different outcome measures: “*suicidal thoughts in the last month, chronic suicidal thoughts in the last month, and suicide attempt in the last year”*; suicidal thoughts were measured using a Likert-like item and affirmative responses were coded dichotomously; to account for chronic suicidal thoughts the same original item was recoded to indicate fleeting suicidal or chronic suicidal ideation; suicide attempt in the last year was measured using the respondent's current age and subtracting the age reported in the item, "How old were you at your last suicide attempt,” and if the result of subtracting was 0, then a 1 was indicated for the binary variable suicide attempt in the last year | Results show that internalized homophobia was associated with two of the suicide outcome variables; a higher rate of internalized homophobia was associated with higher odds of reporting suicidal thoughts in the last month (OR = 1.193, 95% CI = 1.114–1.278, *p* < .001), and reporting chronic suicidal thoughts over fleeting thoughts (OR = 1.271, 95% CI = 1.093–1.479, *p* < .01); as internalized homophobia increases the odds of reporting chronic suicidal thoughts compared to fleeting suicidal thoughts increases (OR = 1.283, 95% C I =1.079–1.525, *p* < .01); however, internalized homophobia was not found to be significantly associated with suicide attempt in the last year |
| Lawlace, 2021^b^ | To examine whether minority stressors predict suicidality | Longitudinal quantitative study with 3 waves of data (baseline, 6-month, and 12-month follow-up) with individuals living in the United States (primarily Illinois), using a non-probability, incentivized snowball sampling approach from various venues (i.e., sexual gender minority community organizations, health fairs, high school/college groups, and social media advertisements), drawn from a larger study with only participants assigned female at  birth (FAB 400; see Dyar et al., 2020 and Dyar et al., 2021) | 369 individuals assigned female at birth, ages 16-32 years; *M* = 19.72, *SD* = 3.36  **Sexual orientation:** Gay or lesbian (*n* = 85, 23%); bisexual or pansexual (*n* = 211, 57.2%); queer (*n* = 42, 11.4%); unsure/questioning (*n* = 16, 4.3%); straight/heterosexual (*n =* 4, 1.1%,); asexual (*n* = 7, 1.9%); not listed (*n* = 4, 1.1%)  **Gender identity:** Cisgender women (*n* = 280, 75.9%); cisgender men (*n* = 12, 3.3%,); transgender (*n* = 13, 3.5%); gender non-conforming (*n* = 22, 6.0%,); genderqueer (*n* = 23, 6.2%); non-binary (*n* = 13, 3.5%); not listed (*n* = 6, 1.6%)  **Racial or ethnic identity:** Black (*n* = 148, 40.1%); Latino/a/e (*n* = 108, 29.3%); White (*n* = 113, 30.6%)  **Other demographics collected**: Researchers also collected information on economic stress, and family income at baseline; majority of family income ranged from $20,001-$70,000 (*n* = 198, 53.6%) | Referred to as *“internalized stigma”*; participants completed The Desire to Be Heterosexual subscale of the internalized stigma measure put forth by another publication from Puckett et al., 2017^c^ (see Sarno et al., 2020 for description); scores were computed by taking the mean of the responses (α = 0.88) | Suicide risk was referred to with “*suicidality outcomes”* (i.e., suicidal ideation, suicide plan, suicide attempts); participants responded to three suicidality items at each wave; to assess suicidal ideation: Participants answered the question, “During the past 6 months, did you ever seriously consider attempting suicide?”; for suicide plan participants answered the question, “During the past 6 months, did you make a plan about how you would attempt suicide?”; for suicide attempt participants answered the question, “During the past 6 months, how many times did you actually attempt suicide?” and responses were then dichotomized to zero times or at least once | Results with bivariate associations show that baseline internalized stigma significantly predicted suicidal ideation over the 12-month follow-up period (OR = 1.56, *p* < .05), but not significantly associated with having a suicide plan over the next year (OR = 1.34, *p* > .10) or attempting suicide (OR = 0.92, *p* > .10); however, when the model controlled for baseline suicidality and demographic controls, the relationship between internalized stigma and suicidal ideation became non-significant |
| Puckett et al., 2017^a^ | Same as Puckett et al., 2017 in Appendix D | Same as Puckett et al., 2017 in Appendix D | Same as Puckett et al., 2017 in Appendix D | Same as Puckett et al., 2017 in Appendix D | Suicide risk was measured through “*suicide attempts”*; youth were asked how many times they had attempted suicide in their lifetime, and this variable was transformed to compare individuals who had never attempted suicide to those who had attempted suicide at least once; those who reported past suicide attempts were asked age of attempt, how they attempted, and about whether the attempt was not related, somewhat related, or highly related to being lesbian, gay, or bisexual | Results indicate that internalized heterosexism alone was not significant in predicting suicide attempts (*b* = -0.14, *SE* = 0.11, *p* = .18, OR = 0.87); rather, the strongest predictor of reporting attempting suicide was whether participants had lost friends after coming out as a sexual minority |
| ^a^ This study examined both depressive and suicide risk outcomes, and is the same study cited in Appendix D (*n* = 1)  ^b^ Indicates grey literature (i.e., master’s thesis or doctoral dissertation) | | | | | | |
